# Mechanisms underlying speech sound discrimination and categorization in humans and zebra finches

**DOI:** 10.1007/s10071-018-1165-3

**Published:** 2018-02-12

**Authors:** Merel A. Burgering, Carel ten Cate, Jean Vroomen

**Affiliations:** 1 Department of Cognitive Neuropsychology, Tilburg University, Warandelaan 2, P.O. Box 90153, 5000 LE Tilburg, The Netherlands; 2 Institute Biology Leiden (IBL) Leiden University, P.O. Box 9505, 2300 RA Leiden, The Netherlands; 3 Leiden Institute for Brain and Cognition (LIBC), Leiden University, Leiden, The Netherlands

**Keywords:** Categorization, Speech perception, Comparative cognition, Songbirds, Zebra finch, Human

## Abstract

Speech sound categorization in birds seems in many ways comparable to that by humans, but it is unclear what mechanisms underlie such categorization. To examine this, we trained zebra finches and humans to discriminate two pairs of edited speech sounds that varied either along one dimension (vowel or speaker sex) or along two dimensions (vowel and speaker sex). Sounds could be memorized individually or categorized based on one dimension or by integrating or combining both dimensions. Once training was completed, we tested generalization to new speech sounds that were either more extreme, more ambiguous (i.e., close to the category boundary), or within-category intermediate between the trained sounds. Both humans and zebra finches learned the one-dimensional stimulus–response mappings faster than the two-dimensional mappings. Humans performed higher on the trained, extreme and within-category intermediate test-sounds than on the ambiguous ones. Some individual birds also did so, but most performed higher on the trained exemplars than on the extreme, within-category intermediate and ambiguous test-sounds. These results suggest that humans rely on rule learning to form categories and show poor performance when they cannot apply a rule. Birds rely mostly on exemplar-based memory with weak evidence for rule learning.

**Electronic supplementary material:**

The online version of this article (10.1007/s10071-018-1165-3) contains supplementary material, which is available to authorized users.

## Introduction

Many studies have demonstrated that nonhuman animals (hereafter: animals) can be taught to discriminate human speech sounds. For example, speech discrimination in Japanese quail (Kluender et al. 1987), pigeons and blackbirds (Hienz et al. 1981), rats (Eriksson and Villa 2006), cats, monkeys (Dewson 1964), budgerigars (Dooling and Brown 1990), ferrets (Bizley et al. 2013), baboons (Hienz and Brady 1988), chinchillas and macaques (Kuhl and Miller 1975; Kuhl and Padden 1982) seems in many ways comparable to that of humans with respect to forming speech sound categories. Recent studies demonstrated that also zebra finches can discriminate isolated vowels and natural or synthetic syllables that differ in vowel (Kriengwatana et al. 2015a; Ohms et al. 2010, 2012). Furthermore, the birds were able to maintain this discrimination when the syllables were pronounced by new speakers of the same sex or the other sex, which reveals the ability to generalize perceptually learned sounds to other speakers (Kriengwatana et al. 2015a; Ohms et al. 2010). However, what type of cognitive mechanisms underlie this discrimination and generalization and to what extent zebra finches can show categorization is yet unknown. Comparative studies can reveal more about the cognitive mechanisms used by birds and humans (Mercado et al. 2005). Here, we compare speech sound categorization of zebra finches and humans using two one-dimensional stimulus–response (SR) mappings in which subjects had to discriminate either ‘*wet’* from ‘*wit’* or male from female speakers, and two two-dimensional SR-mappings in which subjects were required to use both dimensions. After subjects had learned to accurately categorize the trained sounds, we tested generalization to more and less extreme versions of the stimuli. Different theories on the mechanisms underlying categorization predict differences in learning speed between one- and two-dimensional mappings as well as in generalization to novel stimuli (Smith 2014; Smith et al. 2011, 2012, 2016).

Auditory categorization is a cognitive mechanism crucial for speech perception (Erickson and Kruschke 1998; Francis and Nusbaum 2002; Goudbeek et al. 2009; Holt and Lotto 2010), facilitating both first language acquisition in infants (Eimas et al. 1971) and second language acquisition in adults (Holt and Lotto 2006; Kuhl 2004). It allows humans to categorize sounds as being a particular vowel or from a male or female speaker. Categorization involves within-category generalization and between-category discrimination. Categorization also implies mapping of these sounds to an auditory category in a multi-dimensional space (Erickson and Kruschke 1998; Hazan and Barrett 2000). This mechanism is remarkable since categories may overlap and variability within categories may be high (Goudbeek et al. 2009; Hillenbrand et al. 1995). An example of such overlapping categorizations is that for vowels and speaker sex. Both vowel categorization and speaker sex categorization (often described as gender categorization) have been demonstrated in humans (Fuller et al. 2014; Goudbeek et al. 2009; Holt and Lotto 2010; Massida et al. 2013; Skuk et al. 2015). Vowel perception requires both speaker normalization and categorization based on segmental information, mostly determined by the ratio between the two lowest formant frequencies: F1/F2 (Johnson 1990; Kriengwatana et al. 2015b; Polka and Bohn 2003). For categorization based on speaker sex, human listeners mostly rely on the pitch (fundamental frequency—F0) (Fuller et al. 2014; Skuk et al. 2015). Whether and how birds can categorize speech sounds by speaker sex is, to the best of our knowledge, unknown.

The formation of human vowel categories is affected by learning (Kuhl 2004). The exposure to individual sounds results in an abstract representation beyond the exemplars. Different mechanisms may underlie such categorization, such as prototype learning, rule-based learning, or information-integration (Ashby and Maddox 2005; Erickson and Kruschke 1998; Maddox and Ashby 2004; Minda and Smith 2001; Smith et al. 2011, 2012, 2016; Smith and Minda 1999). Such learning mechanisms contrast with exemplar-based memorization, in which sounds in a stimulus set are discriminated based on learning the individual training stimuli. This can be seen as a nonanalytic way of learning (Smith et al. 2012). Generalization to new sounds is then based on the similarity to any of the trained stimuli. With prototype learning, some features of training sounds belonging to the same category are ‘averaged’ to form a prototype. The response to new stimuli depends on the characteristics shared with the category prototypes. Rule-based learning involves the learning of a one-dimensional rule (vowel or speaker sex) or conjunction rule (e.g., press left if stimulus is ‘0’ on dimension *x* and ‘1’ on dimension *y* (‘01’) vs. press right if stimulus is ‘1’ on dimension *x* and ‘0’ on dimension *y* (‘10’)) (Ashby and Maddox 2005). Here, the subjects identify the dimension or combination of dimensions on which stimuli can be distinguished. This analytical learning result in learning a rule that humans can describe verbally. This will lead to optimal categorization if, for example, the pitch of a sound is above or below a certain value (Smith et al. 2011). Information-integration concerns an implicit mechanism that is used when only the integration of two or more dimensions enables correct classification (Gottwald and Garner 1972; Goudbeek et al. 2009; Posner and Keele 1968). Previous studies on visual and auditory categorization showed that humans use a rule-based mechanism, when possible (Goudbeek et al. 2007, 2009; Smith et al. 2012, 2016).

**Fig. 1:**
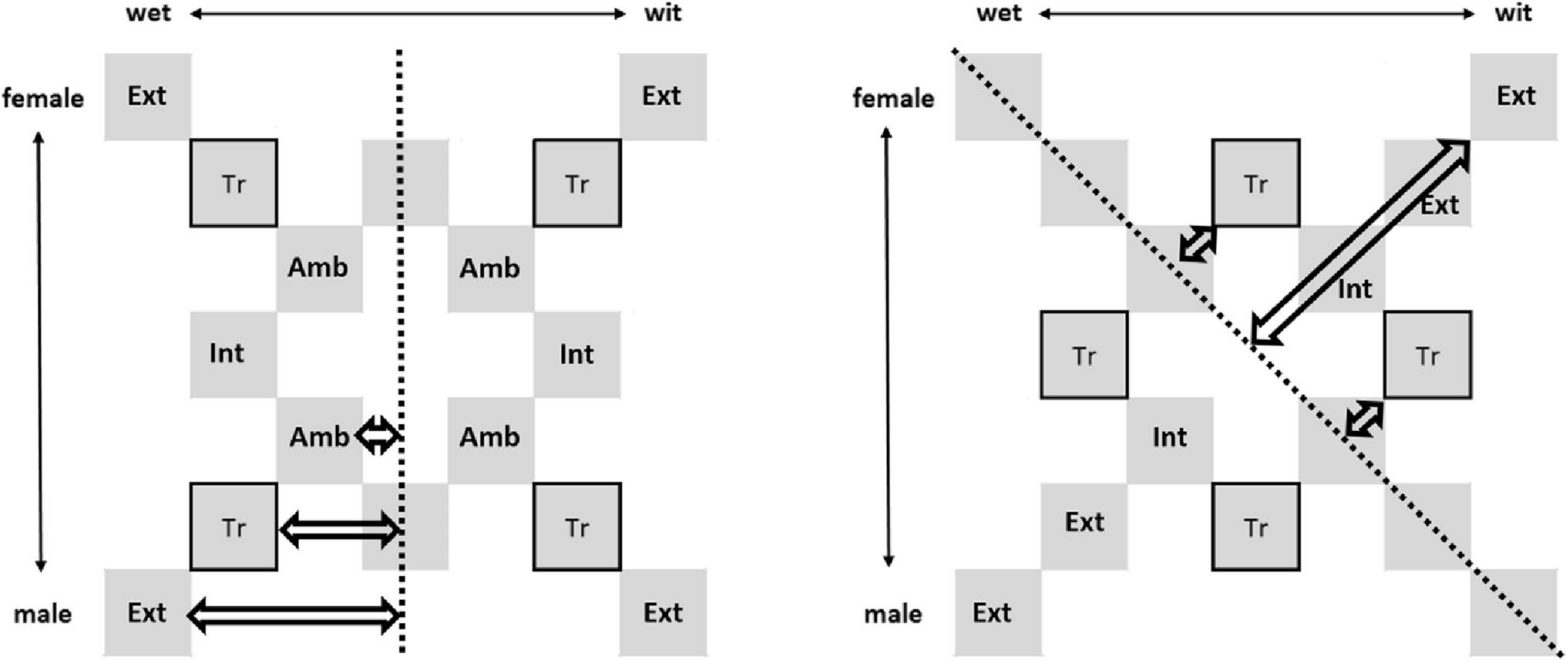
The panels display a stimulus matrix in which the vowel continuum is represented on the *X*-axis, from *wet* to *wit* from left to right, and the speaker sex continuum is represented on the *Y*-axis, from female to male from top to bottom. The distances from the hypothetical category boundary (dashed line) to the trained sounds (Tr), the more extreme (Ext) and more ambiguous (Amb) test-sounds are represented with arrows. If a subject uses exemplar memorization, it will perform best on Tr during the test. If a subject forms categories, generalization to new test-sounds may depend on the distance of these sounds from the category boundary, but one might expect similar responses to Tr, intermediate (Int) and Ext stimuli and possibly a lesser response to Amb. Left panel—example of a one-dimensional mapping (the *wet*–*wit* distinction). Right panel—example of a diagonal mapping. Gray boxes on the dotted lines represent sounds (between categories) not used for the analyses described here

In the current study, we examined the occurrence of processes resulting in category-level knowledge for both humans and zebra finches. We used a 2-alternative forced-choice (2-AFC) task to compare performance on four different speech sound mappings. In one-dimensional mappings, subjects were trained to categorize four training sounds either based on vowel or speaker sex. In addition, there were two two-dimensional mappings, which required the use of both dimensions to classify the stimuli. In one of these mappings, the optimal boundary was diagonal; therefore, we used the descriptive term diagonal mapping (experiment 2) (Ashby et al. 2002). Here, category formation is possible by integrating both dimensions. The other two-dimensional mapping, an exclusive-or (XOR) mapping (Anderson et al. 2006; Ashby et al. 2002; Smith 2014; Smith et al. 2016), also required the combination of vowel and speaker sex, but there is no straight forward rule that could define categories, and subjects had to remember that male *wit* and female *wet* were one category, and female *wit* and male *wet* the other (experiment 3) (Fig. 1). Training continued until criterion was reached. In a subsequent test phase, we examined categorization of the trained sounds by examining generalization to new test-sounds that were either further away from the hypothetical category boundary (extreme test-sounds), closer to the category boundary (ambiguous test-sounds), or in-between the trained sounds (within-category intermediate test-sounds).

We expected humans to have no difficulty with one-dimensional mappings as these may fit already fine-tuned categories for vowels and for speaker sex (Goudbeek et al. 2009). Furthermore, we expected generalization to new test-sounds to depend on the distance of these sounds from the category boundary (Fig. 1). When the categories are well established, the extreme and within-category intermediate test-sounds should be easy to categorize because they are away from the boundary, whereas the ambiguous test-sounds may be harder to categorize because they are close to the boundary. Although zebra finches showed that acoustic differences between different vowels can be more salient than the differences between same vowels produced by different (male) speakers (Dooling 1992), zebra finches obviously do not already possess the human categories for vowels and speaker sex. As a result they might learn to respond to individual training stimuli by exemplar-based memorization. However, during training they might discover acoustical similarities between stimuli and hence also categorize these in a rule-based way. If they use exemplar-based memorization, we then expected performance at test to be best on the trained sounds; generalization to new sounds should depend on the acoustical distance from the trained sounds (Fig. 1). A previous study that demonstrated vowel categorization in European starlings suggests that extensive training on dense vowel distributions with many exemplars is a prerequisite for category learning (Kluender et al. 1998). However, recent studies suggested that zebra finches might acquire categories during training on a small set of stimuli (Kriengwatana et al. 2015a; Ohms et al. 2010). If this is rule-based, then zebra finches’ performance in the test phase should show a human-like pattern of generalization: high performance on the trained, extreme and within-category intermediate test-sounds, but low performance on the ambiguous ones.

In the two-dimensional SR-mappings, subjects were trained to categorize the four training sounds along two dimensions rather than one. We expected humans to have more difficulty learning and maintaining these two-dimensional mappings than the one-dimensional mappings (Goudbeek et al. 2007), in particular for the XOR mapping for which the categories are heterogeneous and allow no generalization. If zebra finches are able to acquire similar dimensions during training to humans, then they might also have more difficulty with the two-dimensional than one-dimensional mappings. Alternatively, if zebra finches are purely relying on exemplar-based categorization, it may not matter if the training sounds vary in one or two dimensions, so performances in test is expected to be similar for all four mappings.

## Methods

### Subjects

*Birds*—We used thirty-six adult zebra finches, (*Taeniopygia guttata)* (18 males and 18 females) from the Leiden University breeding colony. All birds were between 120 and 563 days post-hatching at the start of the experiment. Prior to the experiment, birds were housed in single-sex groups of no more than fifteen animals and they were kept on a 13.5 L:10.5 D schedule at 20–22 °C. The birds always had access to a seed mixture (42% yellow millet, 22% canary seed, 16% yellow panis, 12% white millet, 6% red millet and 2% red panis). Twice a week, the birds received some egg food (mashed boiled eggs) and vegetables and fruits (grated carrots and apple). During the experiment, drinking water, cuttlebone, and grit were available ad libitum. The birds had no previous experience with similar behavioral experiments. All animal procedures were approved by the Leiden Committee for animal experimentation (DEC) (DEC number 14178).

*Humans*—Sixty students from Tilburg University (39 women, 21 men) with mean age of 21 (standard deviation (SD) = 3 years) participated after having given written informed consent. Participants reported normal hearing and were naïve to sounds used in the experiment and research question. All participants received course credits for participation. The study was conducted in accordance with the ethical standards of the 2013 Declaration of Helsinki.

### Apparatus

*Birds*—Zebra finches were individually housed in an operant conditioning chamber (Skinnerbox) (70 (l) × 30 (d) × 45 (h) cm), constructed of wire mesh front and side walls and a foamed PVC back wall. The cage was placed in a sound-attenuated chamber. A fluorescent lamp (Phillips Master TL-D 90 DeLuxe 18 W/965, The Netherlands) served as the light source and was placed on top of the Skinnerbox. The same light/dark schedule as in the breeding colony was applied. The back wall of the cage contained three horizontally aligned gray round pecking keys (hereafter: sensors) with a red LED light at the top of each sensor. Sound stimuli were played at approximately 70 dB (SPL meter, RION NL 15, RION) through a speaker (Vifa MG10SD09-08) 1 m above the cage. The three pecking sensors, the fluorescent lamp, the food hatch and speaker were connected to an operant conditioning controller that also registered all sensor pecks of the bird (supplement Fig. 1). Pecking the middle sensor elicited a sound stimulus and illuminated the LED light of the left and right sensor. Depending on the sound, the bird had to the peck left or right sensor. A correct response resulted in access to food for 8–10 s and an incorrect response led to 1–15 s darkness depending on the experimental phase.

*Humans*—The experiment took place in a dimly lit sound-attenuated room. Instructions were presented on a 19-in monitor positioned at eye-level, 70 cm from the participant’s head. The sound was presented through Sennheiser HD-203 headphones with a peak intensity of 60 dB. The participant responded by pressing one of two buttons on a response box standing in front of the monitor.

### Stimulus material

We created three versions for all sounds in the stimulus matrix in Fig. 1 (hereafter: stimulus matrices). In order to create the three stimulus matrices of morphed speech sounds, recordings of *wet* and *wit* from six speakers (three male, three female) from an earlier study were selected (Ohms et al. 2010). The sound *wet* was pronounced as *wet* in General American English (the open-middle front unrounded vowel in /wεt/ in International Phonetic Alphabet (IPA) and the sound *wit* was pronounced as /*wit*/ in General American English (the near-close near-front unrounded vowel in /wIt/ in IPA). The vowels were chosen based on canonical Dutch F1/F2 values for each sex (Adank et al. 2004). Three stimulus matrices were constructed with Tandem-STRAIGHT (Kawahara et al. 2008; Skuk and Schweinberger 2014), each based on four different natural speech recordings: *wet* and *wit* spoken by one male and *wet* and *wit* spoken by one female. All recordings were selected based on little noise and few fluctuations in the formant frequencies and for each stimulus matrix, recordings were matched based on duration and formants.

**Fig. 2:**
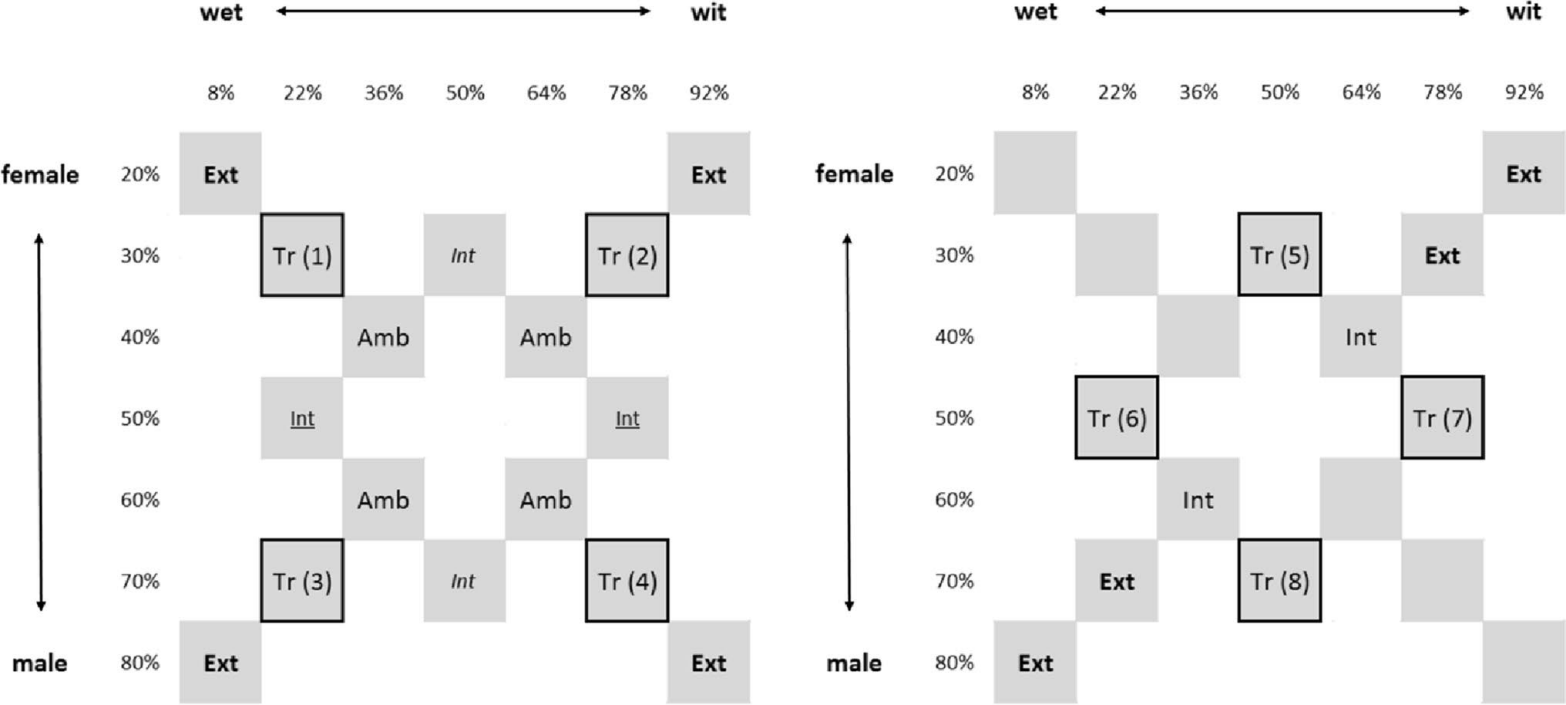
All subjects were trained to categorize four training sounds (Tr1, Tr2, Tr3, and Tr4 for the vowel-, speaker sex-or XOR mapping, and Tr5, Tr6, Tr7 and Tr8 for the diagonal mapping) into two categories. All sounds come from the same set of stimuli but are labeled differently for the vowel-, speaker sex-and XOR mapping versus the diagonal mapping. Upon reaching criterion they were tested on nonreinforced trained and test-sounds. Left panel—The sound labeling for subjects assigned to the vowel-, speaker sex-or XOR mapping. The within-category intermediate sounds for the vowel mapping (Int) and for the speaker sex mapping (*Int*). Right panel—The sound labeling for subject assigned to the diagonal mapping

**Fig. 3:**
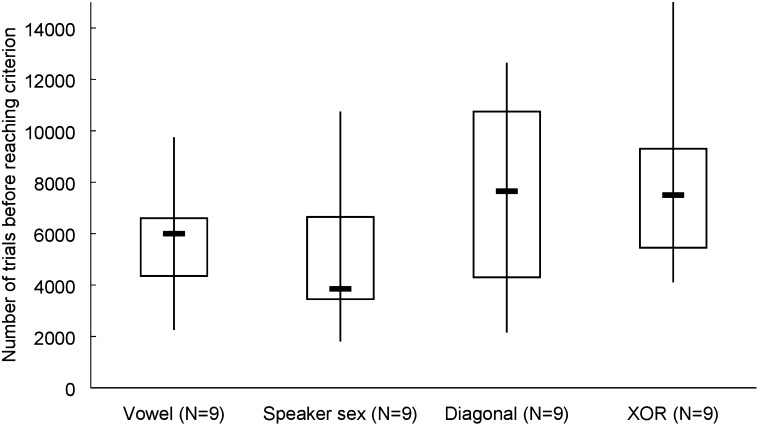
The number of trials for four experimental conditions for the zebra finches. Boxplots display median, interquartile range and full range

**Fig. 4:**
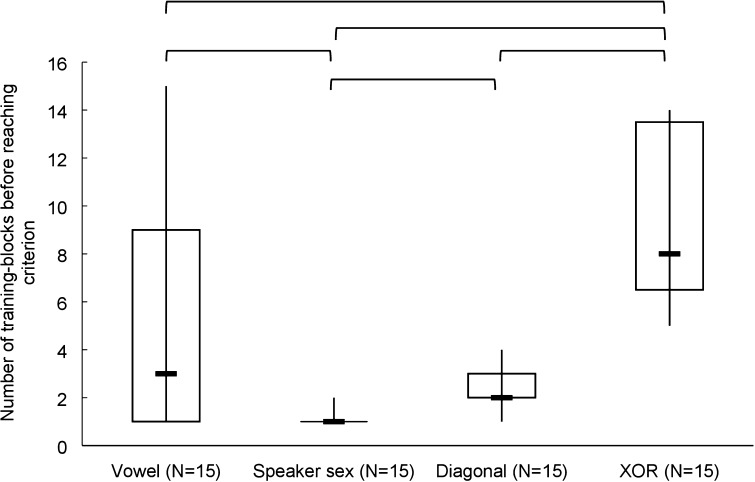
The number of training blocks for four experimental conditions for humans. Boxplots display median, interquartile range and full range. Significant differences (*p* < 0.05) are indicated at the top

Stimulus creation started by creating two male–female continua, one for *wet* and the other for *wit.* From these two male–female morphs, the vowel morphs were constructed following the same procedure. Discriminability of the morphed sounds to humans was tested in a pilot study (*N* = 7). Based on Spearman–Karber curves for all individuals, a step-size of 14% on the *wet*–*wit* continuum and a step-size of 10% on the female–male continuum were chosen in order to balance the salience on both dimensions. Sounds on the *wet*–*wit* continuum went from 8 to 92%, and sounds on the male–female continuum were going from 20 to 80%. We excluded natural end-points in order to keep the acoustic manipulation for all sounds the same. Four training stimuli (supplement Table 1 and Figs. 2–4) and twelve test stimuli, including more extreme and more ambiguous test-sounds were used for all experiments (Fig. 2, left and right).

### Design

The subjects were randomly assigned to one of the different mappings (mapping was between-subjects). Every mapping was completed by 15 humans and nine birds. Per mapping, each of the three versions of the stimulus matrix was used for five humans and for three birds.

### Procedure

All subjects were trained to categorize four training sounds into two categories. Upon reaching criterion, they were tested on the trained and nonreinforced test-sounds. In the vowel mapping, Tr1 and Tr3 (Fig. 2, left panel) were assigned to one category and Tr2 and Tr4 to the other category (Tr1–Tr3 vs. Tr2–Tr4). In the speaker sex mapping, Tr1 and Tr2 were assigned to one category and Tr3 and Tr4 to the other category (Tr1–Tr2 vs. Tr3–Tr4). In the XOR mapping, Tr1 and Tr4 were assigned to one category, and Tr2 and Tr3 to the other category (Tr1–Tr4 vs. Tr2–Tr3). In the diagonal mapping (Fig. 2, right panel), Tr5 and Tr7 were assigned to one category and Tr6 and Tr8 to the other category (Tr5–Tr7 vs. Tr6–Tr8).

*Birds*—At the start of the experiment, every animal was physically examined to allow monitoring of welfare. During the experiment, the birds were closely monitored. If, for some reason, a bird had not been able to obtain food for 18 h, the food hatch opened automatically. Each experiment consisted of a shaping, a training, a transition and a test phase.

Prior to the experiment, the bird needed to acclimate to the cage and learn where to find food. The food hatch was open and the three LEDs on the pecking sensors were switched on. After a few hours up to overnight, the shaping phase was started by closing the food hatch. During the first shaping phase, the bird had to learn to peck all three sensors. Pecking the middle sensor elicited one of the two unfamiliar zebra finch songs (song A of 58 ms or song B of 94 ms), pecking the left sensor or right sensor elicited song A, respectively, song B and led to opening of the food hatch for 10 s. Birds that did not start pecking spontaneously were trained in sessions by flickering the LEDs on the sensors. Once the bird started pecking all sensors, the second shaping phase was started. In this phase, the bird had to learn to initiate its own trial by pecking the middle sensor first and then respond to the played sound by pecking the left or right sensor. When song A was played, pecking the left sensor resulted in food access whereas pecking the right sensor resulted in a preset time of darkness and vice versa for song B. The birds had a response time of 25 s and a trial ended automatically in case the bird did not respond within this time window. An initial darkness of one second built up to 3 s and ultimately 15 s darkness and 8 s food access time. The inter-trial interval was 2 s.

For every day, the discrimination between the stimuli by each bird was calculated as the proportion of correct responses out of all sounds that birds responded to. After 3 days performing at > 0.75, the bird was transferred to the training phase, during which the bird was trained on four training sounds (Tr1-to-Tr4, or Tr5-to-Tr8) according to the relevant SR-schema the bird was assigned to.

After a bird had learned to associate the four training sounds to the correct sensor (overall discrimination score > 0.75, and a score of > 0.60 for each sensor for three consecutive days), the bird was transferred to the transition phase, during which these four stimuli were not reinforced in 20% of the trials for 1 day. By doing so, the bird was prepared for the test phase. During the test phase, 12 new sounds (other morphs out of the same stimuli set) were introduced. Test-sounds were never reinforced and were randomly interspersed between training sounds. Of all trials, 20% were test-sounds and 80% were training sounds. After 40 repeats of all test-sounds, the experiment was finished and the bird was returned to the aviary.

*Humans*—The human participants were instructed to sort the sounds into two different groups. They were left naïve to the relevant SR-assignment. The experiment consisted of three phases: a familiarization phase, a training phase, and a test phase. In the familiarization phase, all four training sounds were played two times in random order in order to familiarize subjects with the sounds. Hereafter, the training phase followed wherein the participants learned to assign the four training sounds into two categories based on visual feedback (‘correct’ and ‘incorrect’) after each response. In the training phase, all four training sounds were repeated five times (20 sounds per training block) in a random order at 100% reinforcement. The participants were promoted to the next phase if accuracy was on average > 0.75 and > 0.60 per category. If the participant did not reach the criteria, the block was repeated until a maximum of 15 blocks (300 trials). The test phase consisted of four blocks of 80 nonreinforced trials each (five × four training sounds and five × 12 new speech sounds in a random order). After each block, the four training sounds were all two times randomly repeated and reinforced. In a short post-experimental questionnaire, humans were asked to explain how they sorted the sounds.

### Analyses

Both for humans and zebra finches, the response data were recorded as binomial measurements (number of left (‘0’) and right (‘1’) responses). For both species, a proportion ‘correct’ for the different sound types was calculated by taking the average scores of the proportion of correct responses to a particular sound type on each side of the midline between the differentially reinforced stimuli (e.g., taking the average of the proportion of correct pecks to ‘extreme *wit*’ and proportion of correct rejections to ‘extreme *wet*’ for the vowel test). For the birds, the proportions correct for the trained sounds included nonreinforced trials only.

*Training*—We measured the number of training trials (birds) or training blocks (humans) required before reaching the overall proportion correct of > 0.75 as well as discrimination for both left and right of > 0.60 on three consecutive days (birds) or one training block (humans). For both species, the distribution for the number of training trials or training blocks of the four different experimental conditions were checked for normality. Because the datasets were not normally distributed, we submitted both datasets (humans and birds separately) to separate Kruskal–Wallis tests, wherein mapping type was the fixed factor. In order to test whether subjects learned one-dimensional mappings (vowel and speaker sex combined) faster than two-dimensional mappings (diagonal and XOR combined), we ran a separate GLM/Mann–Whitney test wherein dimensionality was the fixed factor.

*Test*—For the analysis of each experimental condition, we calculated the proportion of correct responses per sound type, i.e., for each group of trained, extreme, ambiguous and within-category intermediate sounds (Fig. 2). For each sound type, distributions of all proportions correct were checked for normality. For the one-dimensional mappings, the proportion correct for the four extreme sounds, four trained sounds, four ambiguous sounds and the two within-category intermediate sounds (Fig. 2, left) were submitted to two separate 2 (species: human/bird) × 4 (sound type: trained, extreme, ambiguous, within-category intermediate) ANOVA’s. For the diagonal mappings, the proportion correct for the four extreme sounds, four trained sounds and four within-category intermediate sounds (Fig. 2, right) were submitted to a 2 (species: human/bird) × 3 (sound type: trained, extreme, within-category intermediate) ANOVA. For the XOR mappings, the proportion correct for the four extreme sounds, four trained sounds and four ambiguous sounds (Fig. 2, left) were submitted to a 2 (species: human/bird) × 3 (sound type: trained, extreme, ambiguous) ANOVA. Post hoc analyses were performed when the main analyses revealed significant effects.

## Results

### Training phase

*Birds*—In order to reach criterion, birds required on average 5507 (SD = 2291) trials in the vowel mapping, 4884 (SD = 2890) trials in the speaker sex mapping, 7534 (SD = 4185) trials in the diagonal mapping, and 9012 (SD = 5330) trials in the XOR mapping (Fig. 3). In order to test whether the number of trials before reaching criterion was different between the four experimental conditions, a Kruskal–Wallis test was performed which rendered a Chi-square value of 5238 that was not significant (*p* = 0.155). Additionally, we ran a Mann–Whitney test to compare the number of trials in the one-dimensional mappings (vowel and speaker sex) to the two-dimensional mappings (diagonal and XOR), that indicated birds were slower on the two-dimensional (median = 7576) than on the one-dimensional (median = 4601) mappings (*U* = 97.5 and *p* = 0.040).

*Humans*—Humans required on average 5.13 (SD = 4.64) training blocks to reach criterion in the vowel task, 1.07 (SD = 0.26) training blocks to reach criterion in the speaker sex mapping, 2.33 (SD = 0.98) training blocks to reach criterion in the diagonal mapping, and 9.53 (SD = 3.56) training blocks to reach criterion in the XOR mapping (Fig. 4). In order to test whether the number of training blocks before reaching criterion was different between the four experimental conditions, a Kruskal–Wallis test was performed which rendered a Chi-square value of 36.301 (*p* < 0.001). Post hoc analysis with Mann–Whitney tests with a Bonferroni correction showed that the number of training blocks in the speaker sex mapping (median = 1) was significantly faster than in the vowel mapping (median = 3) (*U* = 41, *p* = 0.001), the XOR mapping (median = 8) (*U* = 0.000, *p* < 0.001) and the diagonal mapping (median = 2) (*U* = 27, *p* < 0.001). The number of training blocks before reaching criterion in the vowel mapping (median = 3) was lower than in the XOR mapping (median = 8) (*U* = 53, *p* = 0.013) but not significantly lower than in the diagonal mapping (median = 2) (*U* = 90.5, *p* = 0.351). The number of training blocks before reaching criterion was significantly lower in the diagonal mapping (median = 2) than in the XOR mapping (median = 8) (*U* = 0.000, *p* < 0.001). Thus, humans learn the speaker sex mapping the fastest and the XOR mapping the slowest. To compare the number of training blocks before reaching criterion on the one-dimensional with the two-dimensional mappings, a Mann–Whitney test was performed that indicated that birds were significantly slower on two-dimensional (median = 4, 5) than on one-dimensional mappings (median = 1) (*U* = 214.5 and *p* < 0.001).

### Test phase

**Fig. 5:**
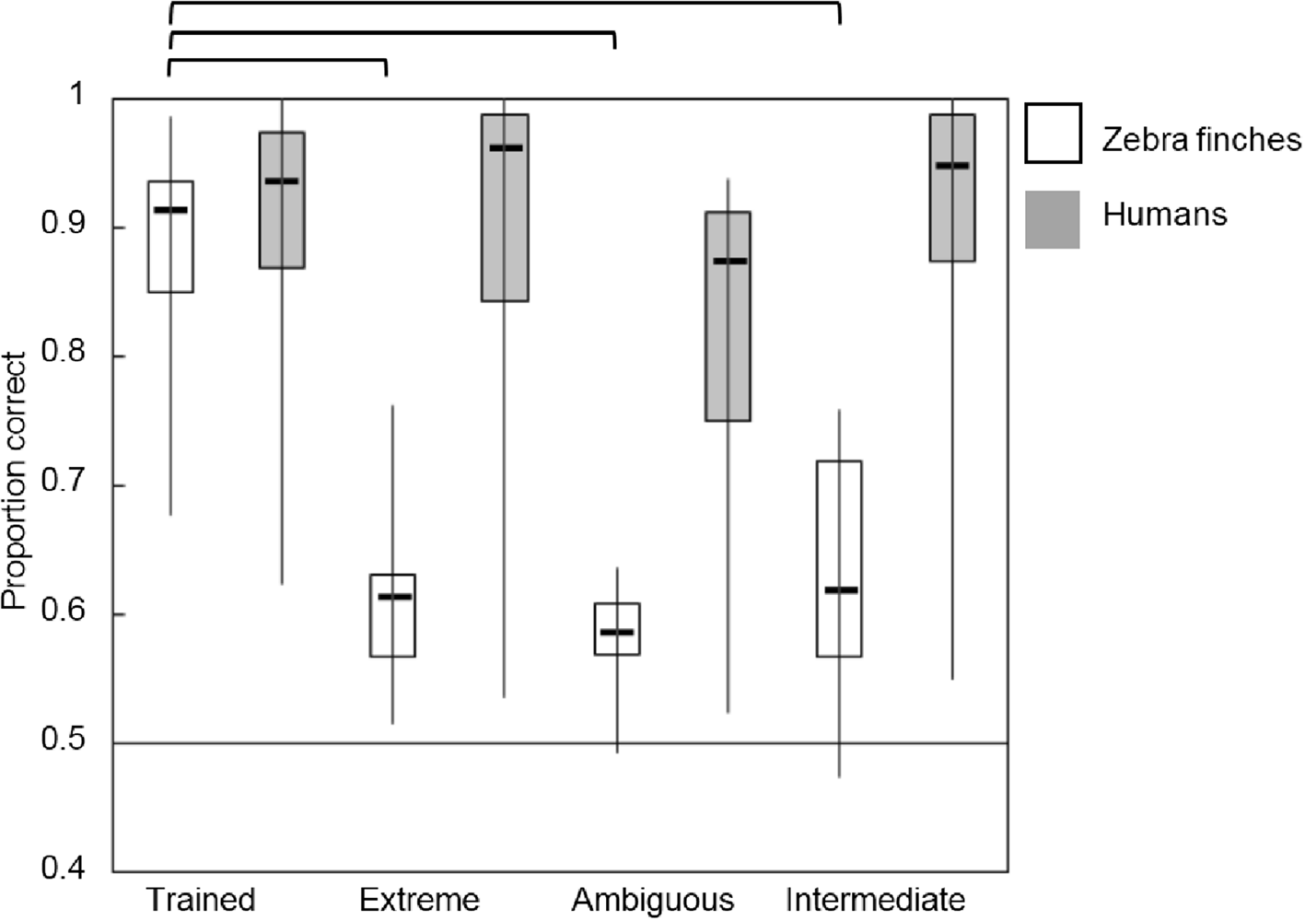
Proportions correct for the four sound type in the vowel mapping. Birds are represented in white, humans in gray. The boxplots represent the median, interquartile range, and full range of the proportions correct for the different sound types. Significant differences (*p* < 0.05) are indicated at the top. The horizontal line marks chance level of 0.5 correct

**Fig. 6:**
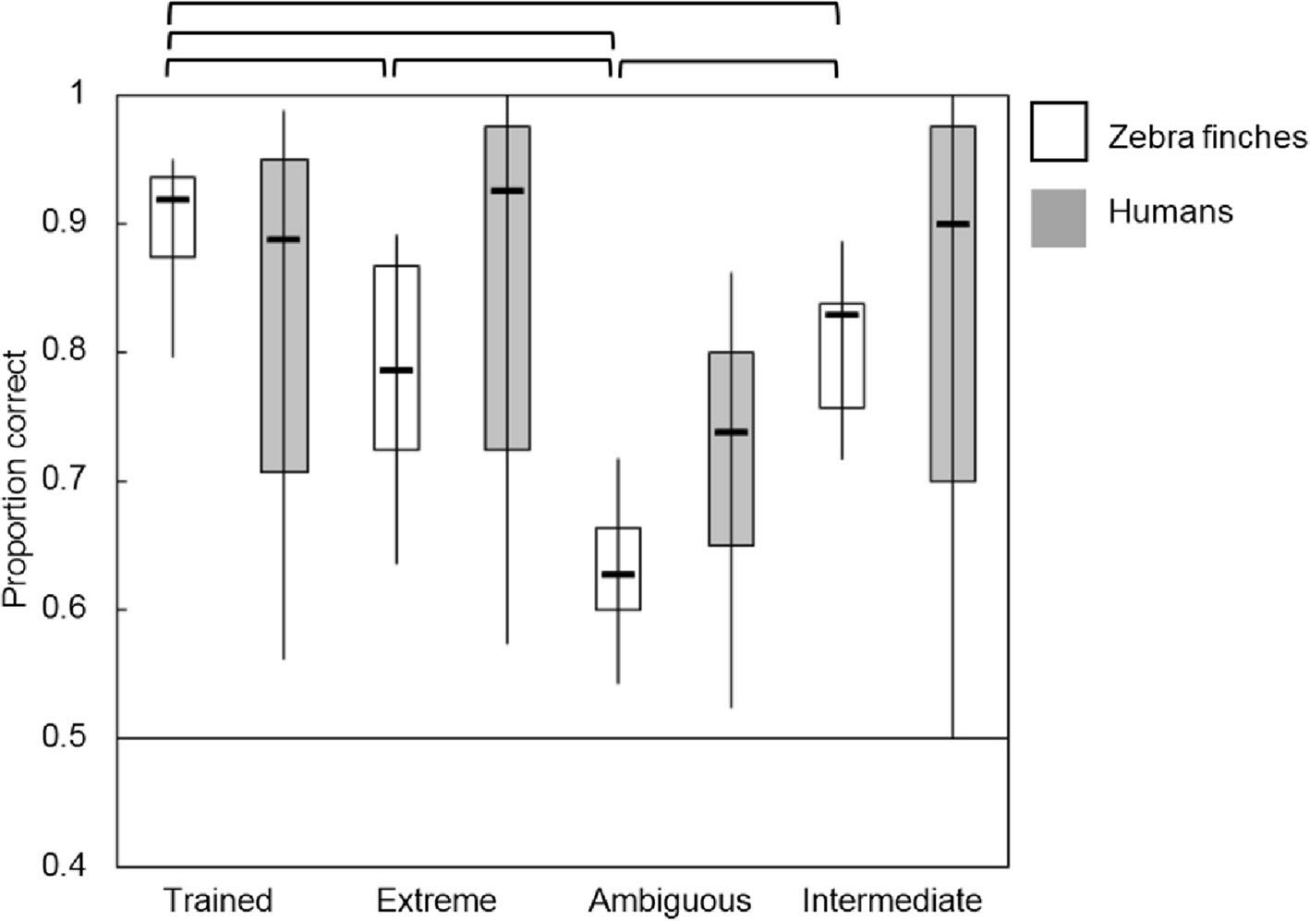
Proportions correct for the four sound types in the speaker sex mapping. Birds are represented in white, humans in gray. The boxplots represent the median, interquartile range, and full range of the proportions correct for the different sound types. Significant differences (*p* < 0.05) are indicated at the top. The horizontal line marks chance level of 0.5

**Fig. 7:**
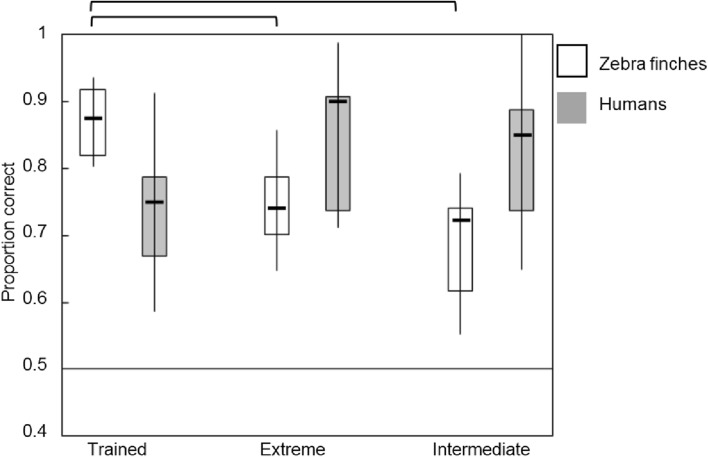
Proportions correct for three sound types in the diagonal mapping. Birds are represented in white, humans in gray. The boxplots represent the median, interquartile range, and full range of the proportions correct for the different sound types. Significant differences (*p* < 0.05) are indicated at the top. The horizontal line marks chance level of 0.5

**Fig. 8:**
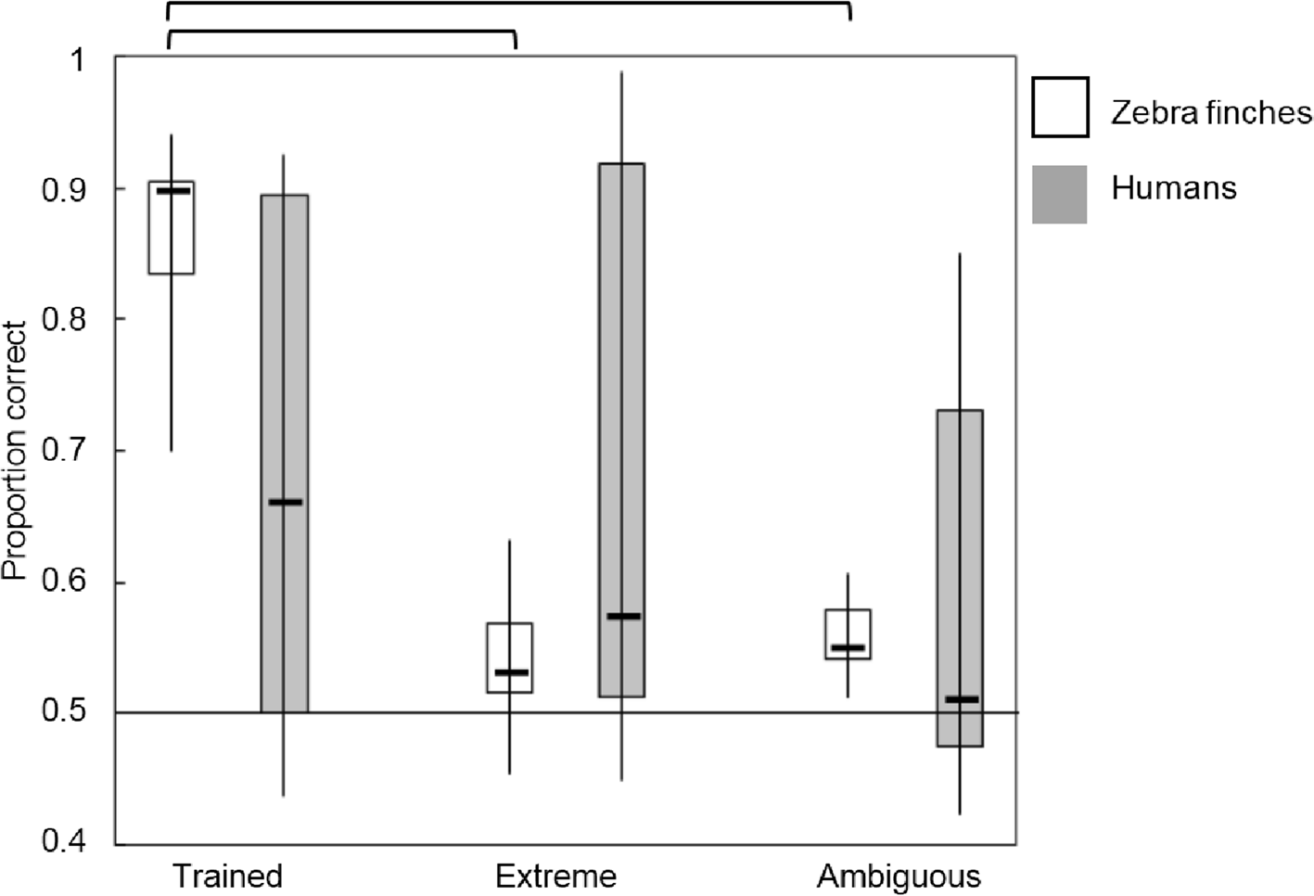
Proportions correct for three sound types in the XOR mapping. Birds are represented in white, humans in gray. The boxplots represent the median, interquartile range, and full range of the proportions correct for the different sound types. Significant differences (*p* < 0.05) are indicated at the top. The horizontal line marks chance level of 0.5

Figures 5, 6, 7 and 8 display boxplots with median, interquartile range and full range of the proportions correct (supplement Tables 2 and 3 for average proportions correct and SD’s). The within-category intermediate sound type is indicated with ‘intermediate.’ We applied an arcsine transformation on the proportion correct because not all were normally distributed. In addition, the supplemental data (Tables 4–7 and Figs. 5–7) contain individual data which suggest the use of different mechanisms among individual birds.

#### Vowel mapping

For individuals trained with the vowel mapping, birds performed much higher on the trained sounds than on the extreme, ambiguous, and within-category intermediate sounds, whereas humans performed about equally high on all sounds. This generalization was supported by a significant main effect for sound type (*F*(3,88) = 9.386, *p* < 0.001) (*η*^2^ = 0.242), species (*F*(1,88) = 72.250, *p* < 0.001) (*η*^2^ = 0.451) and a significant interaction effect for sound type × species (*F*(3,88) = 4.804, *p* = 0.004) (*η*^2^ = 0.141). A one-way ANOVA for birds showed that there was a significant difference between the sound type (*F*(3,32) = 25.497, *p* < 0.001) (*η*^2^ = 0.705). Post hoc analyses showed that proportions correct for trained sounds (0.87 ± 0.09) were significantly higher than extreme (0.61 ± 0.06) (*p* < 0.001), ambiguous (0.58 ± 0.04) (*p* < 0.001) and within-category intermediate sounds (0.62 ± 0.10) (*p* < 0.001). For humans, we found a significant difference for sound type (*F*(3,56) = 2.885, *p* = 0.044) (*η*^2^ = 0.134), but the paired differences were not significantly different from each other in the post hoc analyses. In addition to the analysis at group level, we noted there were individual differences among the birds: one bird out of nine showed generalization to the extreme and within-category intermediate sounds (proportion correct > 0.75), whereas this bird showed a lower proportion correct for the ambiguous sounds (0.62) (supplement Table 4 and Fig. 5). Humans readily generalized to the more and less extreme test tokens. All humans, except one, reported the correct strategy. This person performed on chance level for all new sounds.

#### Speaker sex mapping

For individuals trained with the speaker sex mapping, similar analyses were run as in the vowel mapping. In the 2 (species) × 4 (sound type) ANOVA, there was a main effect of sound type (*F*(3,88) = 8.264, *p* < 0.001) (*η*^2^ = 0.220) indicating that both birds and humans performed relatively low on the ambiguous sounds (birds 0.62 ± 0.05 vs. humans 0.72 ± 0.11) compared to the extreme (birds 0.78 ± 0.08 vs. humans 0.86 ± 0.16), within-category intermediate (birds 0.80 ± 0.05 vs. humans 0.83 ± 0.17) and trained sounds (birds 0.89 ± 0.04 vs. humans 0.83 ± 0.16). There was no effect of species (*F*(1,88) = 3.582, *p* = 0.062) (*η*^2^ = 0.039) and no interaction effect of sound type × species (*F*(3,88) = 1.637, *p* = 0.187) (*η*^2^ = 0.053). In a separate ANOVA for birds, we found a significant difference between sound type (*F*(3,32) = 26.844, *p* < 0.001) (*η*^2^ = 0.716). Post hoc analyses showed that proportions correct for trained sounds were significantly higher than extreme sounds (*p* = 0.001), ambiguous sounds (*p* < 0.001), and intermediate sounds (*p* < 0.004). The extreme versus within-category intermediate sounds did not differ significantly from each other (*p* = 1.000). Proportions correct for ambiguous sounds were significantly lower than extreme sounds (*p* < 0.001) and within-category intermediate sounds (*p* < 0.001). For humans, we found a significant difference between sound types (*F*(3,56) = 3.073, *p* = 0.035) (*η*^2^ = 0.141) but post hoc analyses were all nonsignificant between any combination of two sound types. Individual data showed that five out of nine birds showed generalization to extreme and within-category intermediate sounds (proportion correct > 0.75) (supplement Table 5 and Fig. 6). These birds performed higher on new extreme and within-category intermediate sounds than on ambiguous sounds. Humans readily generalized to the more and less extreme test tokens. Three humans in the speaker sex mapping reported that they categorized the sounds based on vowels instead of speaker sex. The others reported the correct strategy. Individual data showed that four humans performed low (< 0.70 proportion correct) and close to chance level on all sound types.

#### Discussion one-dimensional mappings

In the vowel mapping, zebra finches showed limited generalization to new test-sounds and always performed best on the trained exemplars, a pattern that is indicative of exemplar-based categorization. In the speaker sex mapping, birds also performed best on the trained exemplars, but at the individual level five out of nine birds also showed considerable generalization to extreme and within-category intermediate test-sounds, suggestive of rule-based categorization. Humans showed a generalization pattern that indicates rule-based categorization both for vowels as for speaker sex with higher performance on extreme than ambiguous test-sounds. Both one-dimensional-mappings are easy to solve and verbalize with a simple rule (/e/vs./I/, or male vs. female) and match with human categories for vowel and speaker sex.

### Diagonal mapping

For individuals trained with the diagonal mapping, the 2 (species) × 3 (sound type) ANOVA showed that the main effect of sound type was not significant (*F*(2,66) = 0.921, *p* = 0.403) (*η*^2^ = 0.027) and the main effect of species was also not significant (*F*(1,66) = 0.844, *p* = 0.361) (*η*^2^ = 0.013) (Fig. 7). There was a significant interaction effect for sound type × species (*F*(1,66) = 11.705, *p* < 0.001) (*η*^2^ = 0.262). Birds showed relatively high performance on the trained sounds (birds 0.87 ± 0.05 vs. humans 0.73 ± 0.09) whereas humans performed higher on the extreme (birds 0.74 ± 0.06 vs. humans 0.92 ± 0.09) and within-category intermediate sounds (birds 0.68 ± 0.08 vs. humans 0.82 ± 0.10). In a separate ANOVA for birds, we found a significant difference between sound types (*F*(2,24) = 17.125, *p* < 0.001) (*η*^2^ = 0.588). Post hoc tests showed that performance on the trained sounds was significantly higher than on extreme sounds (*p* = 0.001) and within-category intermediate sounds (*p* < 0.001). Individual data showed that four out of nine birds showed generalization to extreme sounds (proportion correct > 0.75) and two birds showed generalization to within-category intermediate sounds (proportion correct > 0.75) (supplement Table 6 and Fig. 7). The separate ANOVA for humans only found a marginally significant effect of sound type (*F*(2,42) = 3.157, *p* = 0.053) (*η*^2^ = 0.131), revealing that humans performed slightly higher on the extreme and within-category intermediate sounds than on the trained sounds. Five out of fifteen human participants reported that they used the speaker sex dimension to categorize the new sounds and six participants reported that they used the vowel dimension. The others did not report a strategy.

#### Discussion diagonal mapping

Birds showed relatively high performance on the trained sounds, which indicates that they show mostly exemplar-based categorization, with some evidence for use of information-integration in a few birds. Humans performed higher on the extreme and within-category intermediate sounds (although not significant) than on the trained sounds. This outcome may suggest that this mapping induced humans to integrate both dimensions. In the post-experimental questionnaire, 11 out of the 15 humans reported that they categorized the sounds on one dimension, either vowel or speaker sex, thus indicating that they were not able to describe their categorization performance explicitly.

#### XOR mapping

For individuals trained with the XOR mapping, the 2 (species) × 3 (sound type) ANOVA showed a significant main effect for sound type (*F*(2,66) = 9.895, *p* < 0.001) (*η*^2^ = 0.231) and a nonsignificant main effect for species (*F*(1,66) = 0.245, *p* = 0.622) (*η*^2^ = 0.004) (Fig. 8). The interaction between sound type × species was significant (*F*(2,66) = 7.129, *p* = 0.002) (*η*^2^ = 0.178). Birds performed higher on the trained sounds (0.86 ± 0.06) than new sounds (ambiguous: 0.55 ± 0.03 and extreme 0.54 ± 0.05), whereas for humans this difference between sound types was nonexistent. A separate ANOVA for birds confirmed that there was a significant difference between sound types (*F*(2,24) = 87.011, *p* < 0.001) (*η*^2^ = 0.879). Post hoc tests demonstrated that performance on trained sounds was significantly higher than on extreme (*p* < 0.001) and ambiguous sounds (*p* < 0.001). Individual data showed that all birds performed high on trained sounds, pointing toward strong exemplar-based memorization, but they showed a less distinctive pattern for the new test-sounds (supplement Table 7).

The same ANOVA for humans showed that there was no significant difference between the sound types (*F*(2,42) = 1.706, *p* = 0.194) (*η*^2^ = 0.075). Among humans, there was more variation in the proportions correct for all sound types. Individual data showed that six out of fifteen humans performed high (> 0.75 proportion correct) on the trained and extreme sounds whereas five humans performed around chance level. Two participants reported that they used a one-dimensional rule (either vowel or speaker sex) and the others could not describe their strategy.

### Discussion XOR mapping

In the XOR mapping, birds showed much higher performance on the trained sounds than on the extreme or ambiguous sounds. The proportions correct on those sounds are close to chance level. This suggest that zebra finches had formed an exemplar-based memory of the training sounds. Humans had great difficulty with the XOR mapping, presumably because they easily confused the SR-assignment.

## General discussion

Humans and birds were trained to categorize four speech sounds that differed in vowel and speaker sex into different functional categories according to various SR-mappings. Birds showed no significant overall differences in learning the different SR-mappings, whereas humans showed fastest learning in the speaker sex mapping, and slowest learning in the XOR mapping. However, zebra finches did show significantly faster learning when both one-dimensional mappings were taken together and compared with the two-dimensional mappings combined. For humans, this finding fits the hypothesis that in one-dimensional mappings they preferentially rely on preexisting categories that are rule-based, whereas they need to employ a different learning strategy in two-dimensional mappings because they cannot apply a simple rule. For zebra finches, the effect is weaker, but might indicate that they may also be able to use similarities between the stimuli to enhance their learning, and that nonanalytic, exemplar-based processing is not the only system through which birds categorize sounds (Smith et al. 2016). Nevertheless, the birds seem to rely more on exemplar-based memorization than humans. The responses of humans and birds to the test stimuli support these conclusions. Below, we will first discuss the results obtained by the birds in more detail, next those by humans and end with a comparison of both.

*Birds—*Based on previous studies (Ohms et al. 2010, 2012), we expected birds to be able to categorize speech sounds based on vowels, even after training on a small stimulus set. However, generalization in the one-dimensional vowel test was limited, suggesting that exemplar memorization was dominant in the present experiment. An explanation for the discrepancy between the earlier results of Ohms et al. (2010) and the current ones may be the different number of training stimuli. We trained the birds on four-to-two mappings whereas Ohms et al. (2010) trained the birds in the first phase on a two-to-two mapping and in the next phase on ten-to-two mappings (*wet* vs. *wit*, spoken by either five male or five female voices) using a Go-nogo paradigm. During the test phase, we presented the birds with new stimuli, whereas Ohms et al. (2010) used an incremental test setup with a transfer training in which the five voices were replaced by novel voices of either the same or the other sex. Possibly, the more extensive training exposure to different voices by Ohms et al. (2010) might have enhanced vowel category formation. It indicates that our design may not have used sufficient variation with respect to the number of training stimuli per category to induce categorization, and hence may have been biased against obtaining categorization.

In the speaker sex test, birds had higher proportions correct for trained tokens compared to the ambiguous, extreme, and within-category intermediate sounds, suggesting that their memorization was again mainly based on exemplar learning. Nevertheless, extreme and within-category intermediate sounds were better categorized than ambiguous sounds. Also individual data showed that some birds showed clear generalization in the speaker sex mapping, despite the limited number of training stimuli. To our best knowledge, generalization on a speaker sex dimension has not yet been demonstrated in animal research. This indicates the presence of some sort of rule-like learning process, although higher performance on extreme and within-category intermediate sounds compared to ambiguous sounds in a unidimensional mapping does not necessarily imply analytic processing (Wills et al. 2009). It is hard to assess whether high performance on within-category intermediate and extreme sounds is really due to rule learning or normal generalization.

Our findings raise the question which acoustic cues (sound parameters) the birds used in learning the SR-mapping in the one-dimensional tests. The generalization across speakers of both sexes shown in the experiments by Ohms et al. (2010) was ascribed to the birds generalizing on the basis of the formant ratios only, as neither the absolute frequency of the formants nor that of the underlying pitch of the voices (F0) could be used to that end. The bird that generalized in the vowel mapping in this experiment might also have used this feature. In contrast, the generalization in the speaker sex test cannot be based on formant ratios, as the training stimuli for both male and female voices contained the same vowels (/e/ and /I/). In this case, the birds might, similar to what is known from humans, have used the pitch as the most salient factor distinguishing male from female voices. This would imply that zebra finches attend to both and have the flexibility to use either absolute or relative frequency features and to single out one dimension for making generalizations. However, due to our holistic morphing of natural speech sounds, the question which parameters birds used in their discrimination between the sounds remains open.

In the two-dimensional mappings, the birds showed strong memorization of the trained sounds. The generalization for extreme sounds in the diagonal mapping, displayed by four out of nine individuals, suggests that these zebra finches displayed some implicit categorization possibly by integrating information of both dimensions. These results suggest that there is not a single mechanism used by all birds. Individual data of the XOR mapping showed that all birds performed high on trained sounds, pointing toward strong memorization, but they performed just above chance level for the new test-sounds. For this SR-mapping, the birds thus seemed to rely strongly on exemplar-based memorization.

To summarize the results of the zebra finches, it seems that the most prominent mechanism that zebra finches use to generalize to novel speech items is exemplar-based learning. Nevertheless the results of some individuals in the one-dimensional mappings strongly suggest that zebra finches also have the ability to categorize stimuli in a more rule-like manner, while the two-dimensional diagonal mapping shows evidence of information-integration based learning.

*Humans*—Given the evidence for vowel categorization and speaker sex categorization by humans (Goudbeek et al. 2009), as well as their propensity for rule learning (Smith et al. 2012, 2016), we expected humans to have no difficulty with categorization in the one-dimensional SR-mappings. Nevertheless, humans were significantly faster in the speaker sex training. Possibly, humans approach the training at first as a multi-talker environment wherein they try to identify the different speakers (Fuller et al. 2014) before they focus on the (in this case irrelevant) content. Humans showed no clear difference between the SR-mappings in how they generalized to new test-sounds. Nevertheless, three participants in the speaker sex test reported that they categorized the sounds based on vowels. Their strategy in the test phase may be attributed to the fact that feedback was very occasional (Goudbeek et al. 2009), but the fact that we only found a shift from using the speaker dimension toward the vowel dimension and not in the other direction may suggest a bias toward vowel categorization rather than speaker sex categorization.

Since humans are known to initially use a one-dimensional solution in a multi-dimensional SR-mapping they tend to find multi-dimensional mapping harder (Ashby et al. 1999; Goudbeek et al. 2009). We therefore expected that the two-dimensional SR-mappings would be harder to learn than the one-dimensional mappings. Indeed, learning in both the speaker sex and the vowel mapping was faster than in the XOR mapping. Also, the speaker sex mapping was learned faster than the diagonal mapping. The faster learning in the one-dimensional mappings fits the hypothesis that humans use their preexisting categories in these mappings.

In the tests, humans readily generalized in the one-dimensional mappings, as expected based on their preexisting categories. The post-experimental self-reports confirm this inference. Humans also show generalization in the diagonal mapping. They reported often that their categorization was based on a simple rule (vowel or speaker sex) but the high proportions correct for extreme sounds suggested that some people used both dimensions suggesting that they used an implicit information-integration approach that they could not verbalize (Goudbeek et al. 2009). Humans had great difficulty with categorization in the XOR mapping. One participant reported that he approached the XOR mapping as a one-dimensional vowel mapping and another participant reported that she approached the task as a one-dimensional speaker sex mapping, i.e., here also they attempted to apply a unidimensional solution in a multi-dimensional mapping, as has also been reported in other studies (Ashby et al. 1999).

To summarize the data for humans: they demonstrate clear evidence of rule-based categorization in the one-dimensional mappings and the ability to use either the vowel or the speaker sex dimension to categorize speech sounds. When such a rule-based strategy is not possible, humans struggle with categorizing test stimuli although implicit information-integration learning seems present.

*Birds versus humans*—While our findings show that birds seem capable of a limited degree of rule learning and categorization based on an information-integration mechanism, it is also clear that they rely primarily on exemplar-based memorization. There is a considerable gap between their performance and that of humans. For humans, the sharp contrast between the high performance on one-dimensional mappings and the low performance on, in particular, the XOR mapping showed that rule-based categorization is much more developed in humans than in birds. In contrast, the birds are much better at discriminating the training sounds from the test-sounds in the XOR mapping than humans are—a result that indicates that birds can readily use an exemplar-based categorization mechanism, while humans struggle by trying to solve the mapping in a more analytical, rule-based way. While this may reflect a genuine and fundamental species difference in categorization mechanisms, it cannot be excluded that humans’ lifetime exposure to the variety of speech sounds may contribute to the species difference. Also, training the birds with a more extensive set of stimuli in the one-dimensional mappings might have resulted in a clearer evidence of rule-based categorization.

Our findings fit, at least to some extent, visual categorization experiments. In these experiments, involving categorizations somewhat comparable to our auditory experiments, macaques, capuchin monkeys and humans learned a one-dimensional SR-mapping faster than a two-dimensional information-integration mapping (Smith et al. 2012). Pigeons, however, learned these mappings equally quickly (Smith et al. 2012), presumably by using a nonanalytic exemplar-based learning mechanism. This led Smith et al. (2012) to conclude that monkeys, but not pigeons, are capable of more analytical, rule-based like learning. From this they suggest that pigeons may be representative of an ancestral vertebrate categorization system dominated by an integral, holistic and nonanalytic learning mechanism (Smith et al. 2016). However, our zebra finch data provide some evidence that a more analytical and integrative learning can also be present in some bird species. Among birds, species like corvids and some parrots show cognitive abilities at a level comparable to that of primate species (ten Cate and Healy 2017). Also, budgerigars show more evidence of abstraction in an auditory rule learning mapping than zebra finches (Spierings and ten Cate 2016), which in turn seem capable of detecting more regularities in auditory signals than pigeons (ten Cate et al. 2016). For this reason, we suggest that further comparative studies are needed to reveal the phylogenetic distribution and evolution of different types of categorization systems and how and why the differences between various species, including humans, evolved.

## Electronic supplementary material

Below is the link to the electronic supplementary material.

Supplementary material 1 (DOCX 4323 kb)
