# Supplementary material for: Mechanisms underlying speech sound discrimination and categorization in humans and zebra finches
Source: Anim Cogn. 2018 Feb 12;21(2):285–99. doi: 10.1007/s10071-018-1165-3 (PMC5818571; doi:10.1007/s10071-018-1165-3)
Supplement: Supplementary file 1 — Supplementary material 1 (DOCX 4323 kb) [file 10071_2018_1165_MOESM1_ESM.docx]

**Supplementary material**


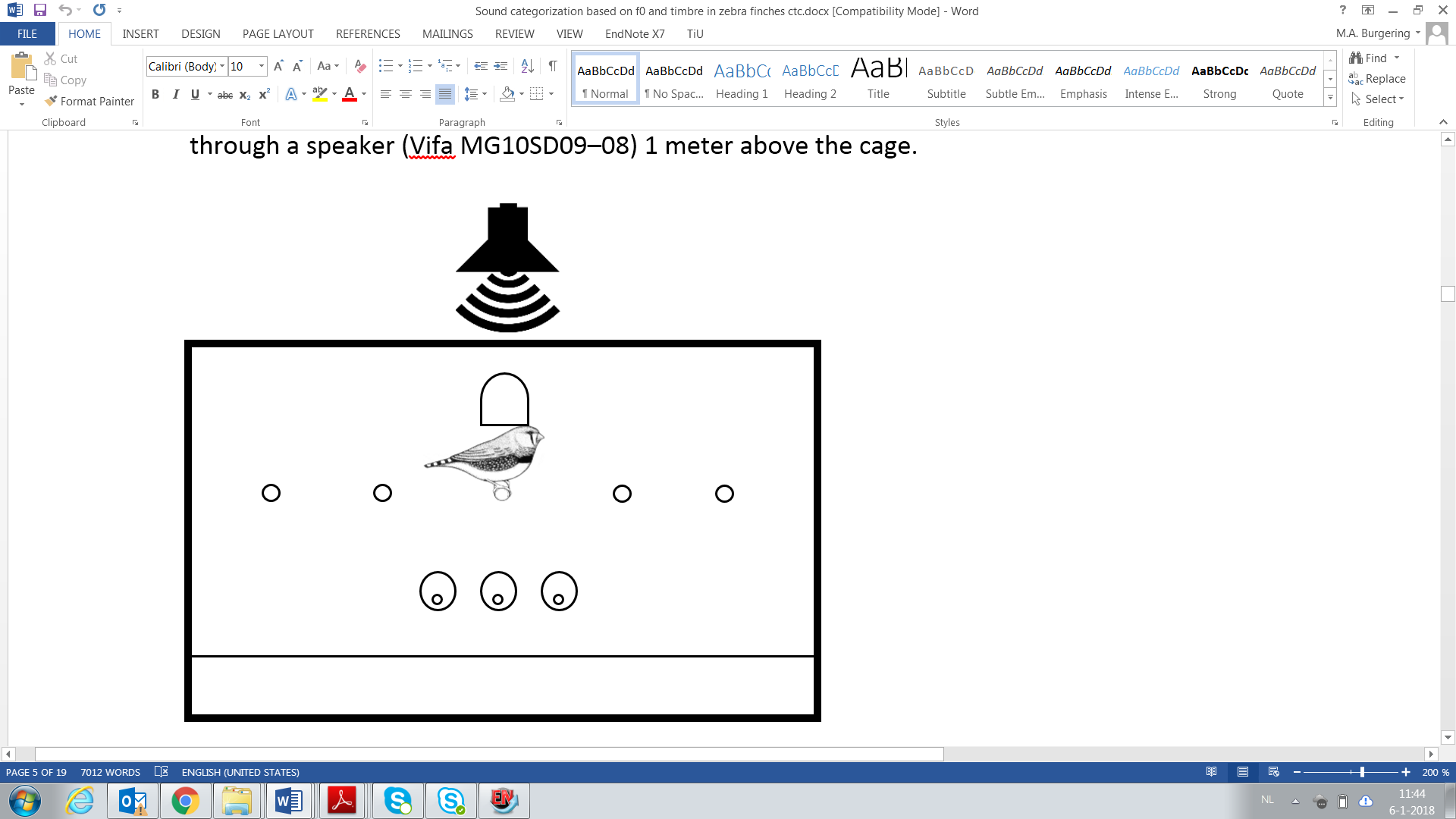


**Fig 1** A schematic picture of the operant conditioning chamber. Three horizontally aligned pecking sensors are depicted at the bottom just above the long horizontal perch. The food hatch was located in the middle of the back wall and accessible from the top middle perch, where the zebra finch is depicted in this figure. When the zebra finch gave the correct response, the food hatch opened (door moved upwards) and the bird was free to eat seed mixture for the allotted time

**Table 1** Center frequencies for formants of training sounds from set 1. Timeframe is approximately 0.1 seconds

| **Stimulus** | **F0** | **F1** | **F2** | **F3** |
| --- | --- | --- | --- | --- |
| Tr1 | 182 | 515 | 2000 | 2755 |
| Tr2 | 185 | 498 | 1867 | 2752 |
| Tr3 | 137 | 450 | 2163 | 2550 |
| Tr4 | 136 | 429 | 2011 | 2999 |
| Tr5 | 183 | 476 | 2072 | 2662 |
| Tr6 | 157 | 516 | 1944 | 2834 |
| Tr7 | 158 | 453 | 2084 | 2704 |
| Tr8 | 136 | 460 | 1944 | 2856 |


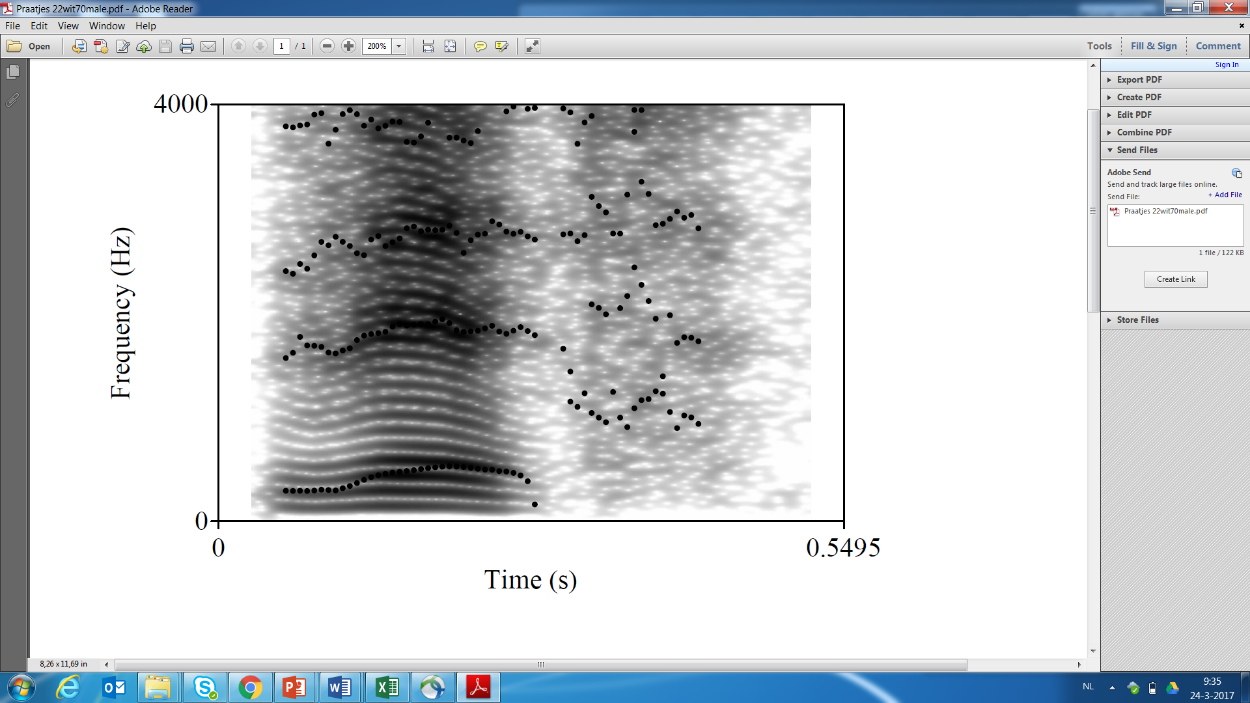
A. Tr1 B. Tr2
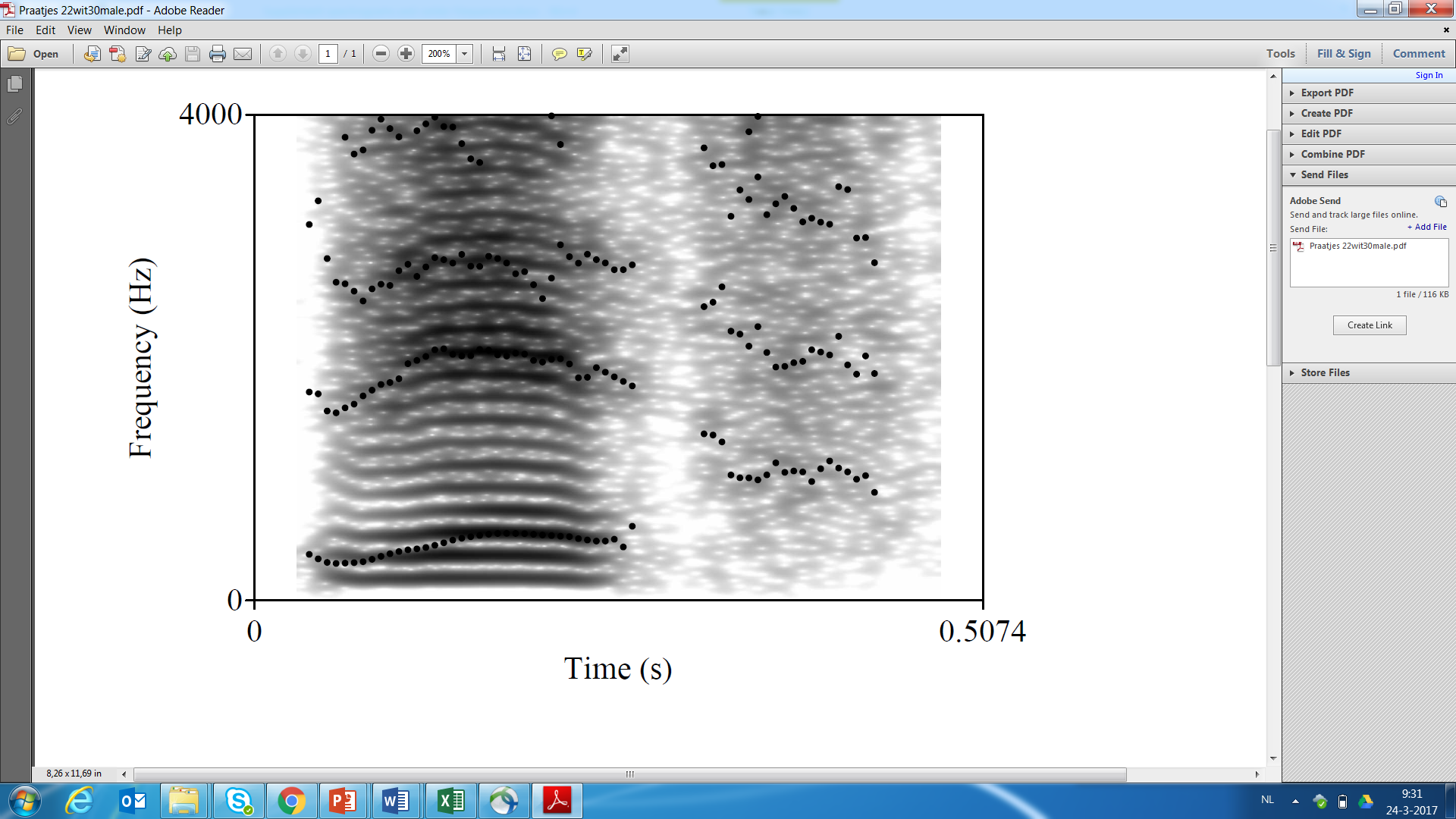


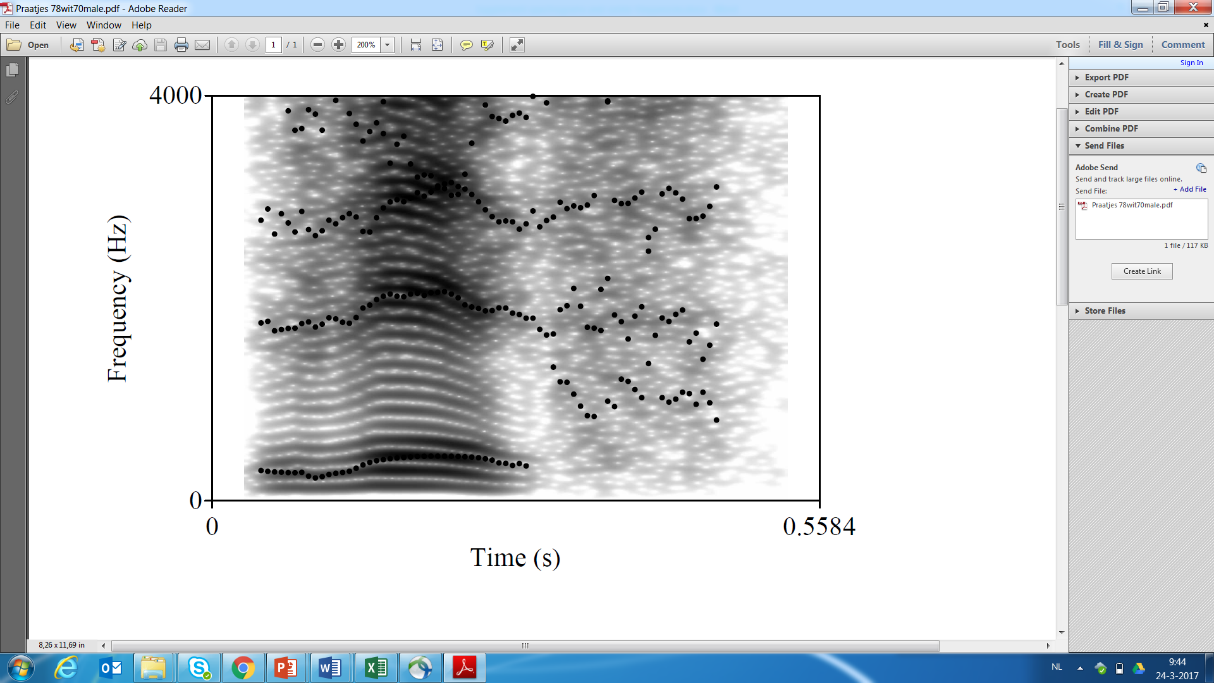
C. Tr3 D. Tr4
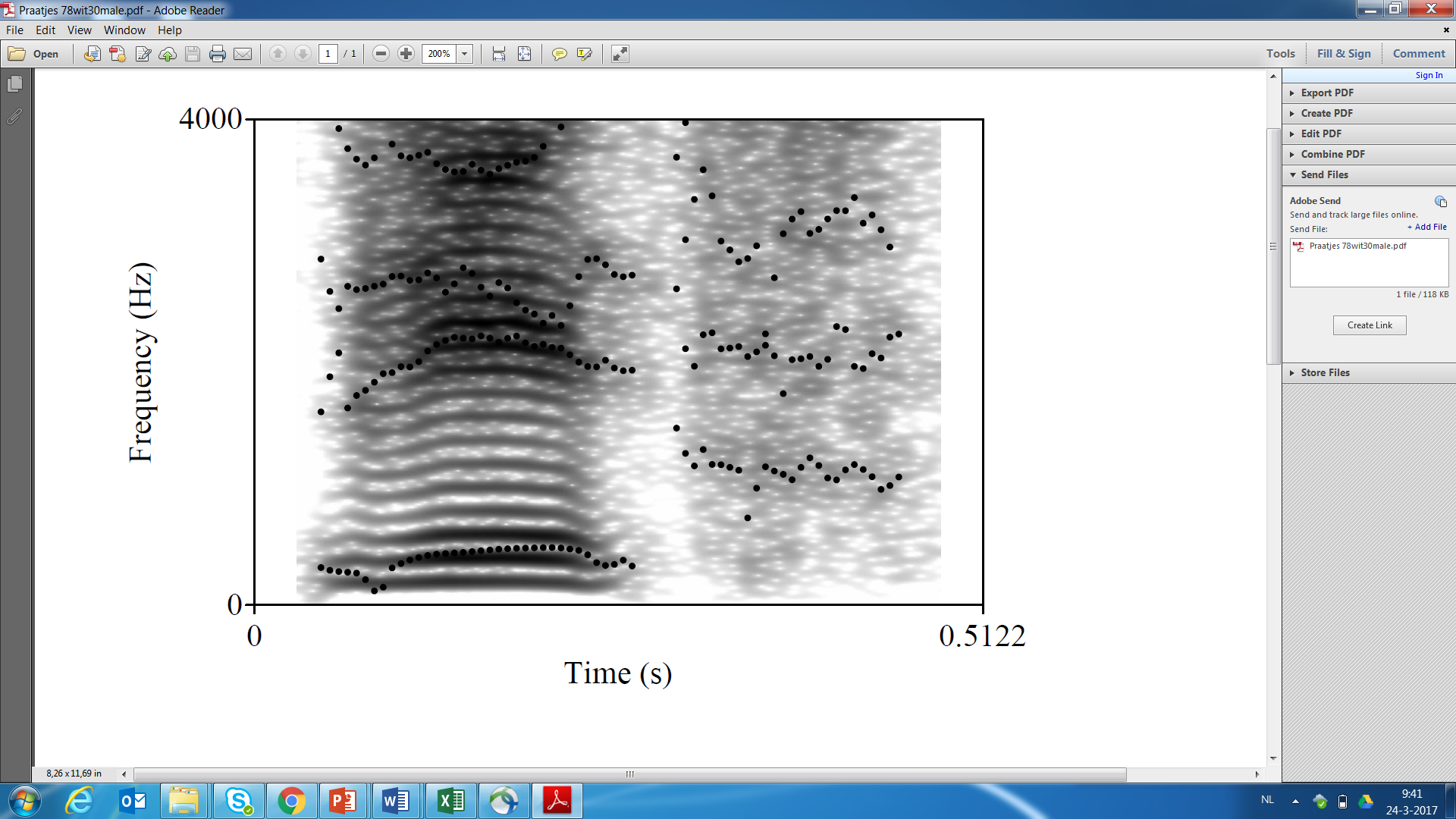


**Fig 2** Spectrograms and the formant trajectories for training sounds (A. Tr1; B. Tr2; C. Tr3; D. Tr4) for the speaker sex, vowel and XOR mapping from set 1


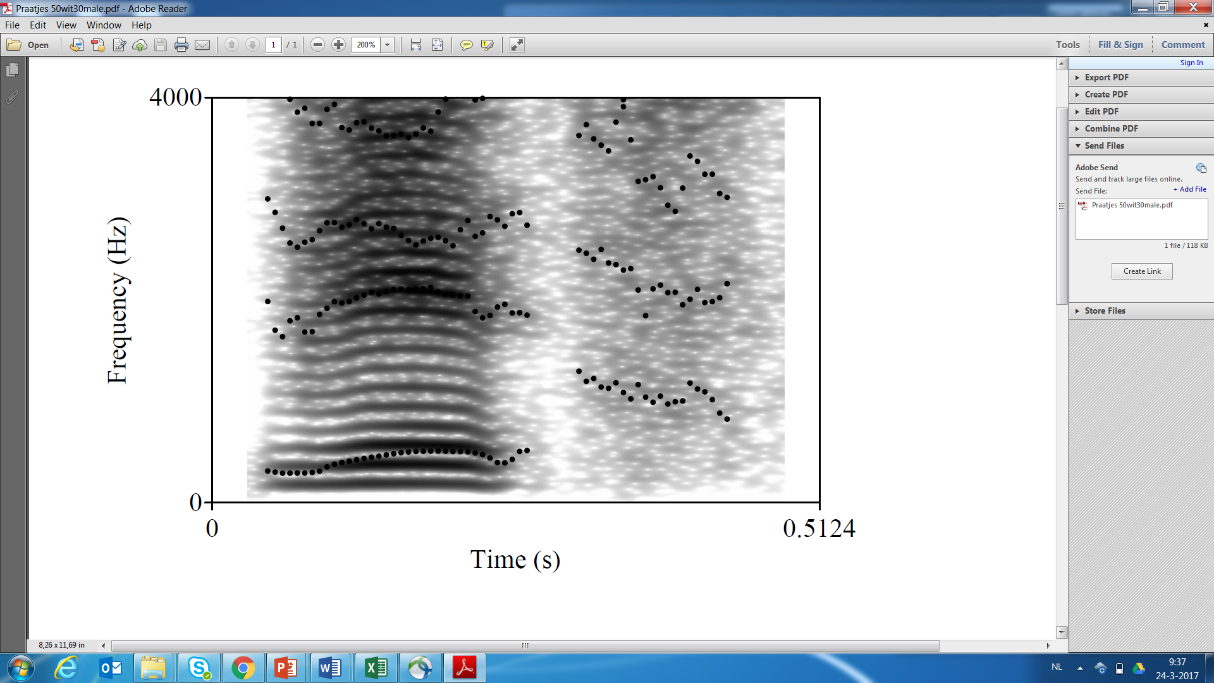

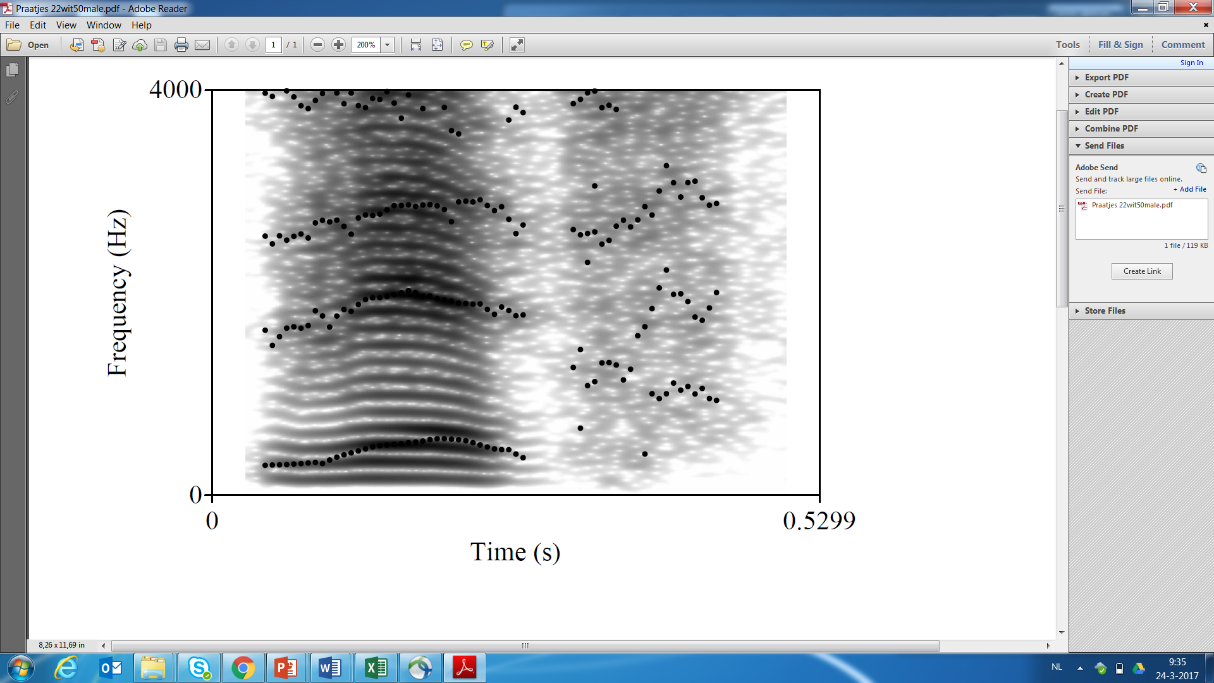
E. Tr5 F. Tr6


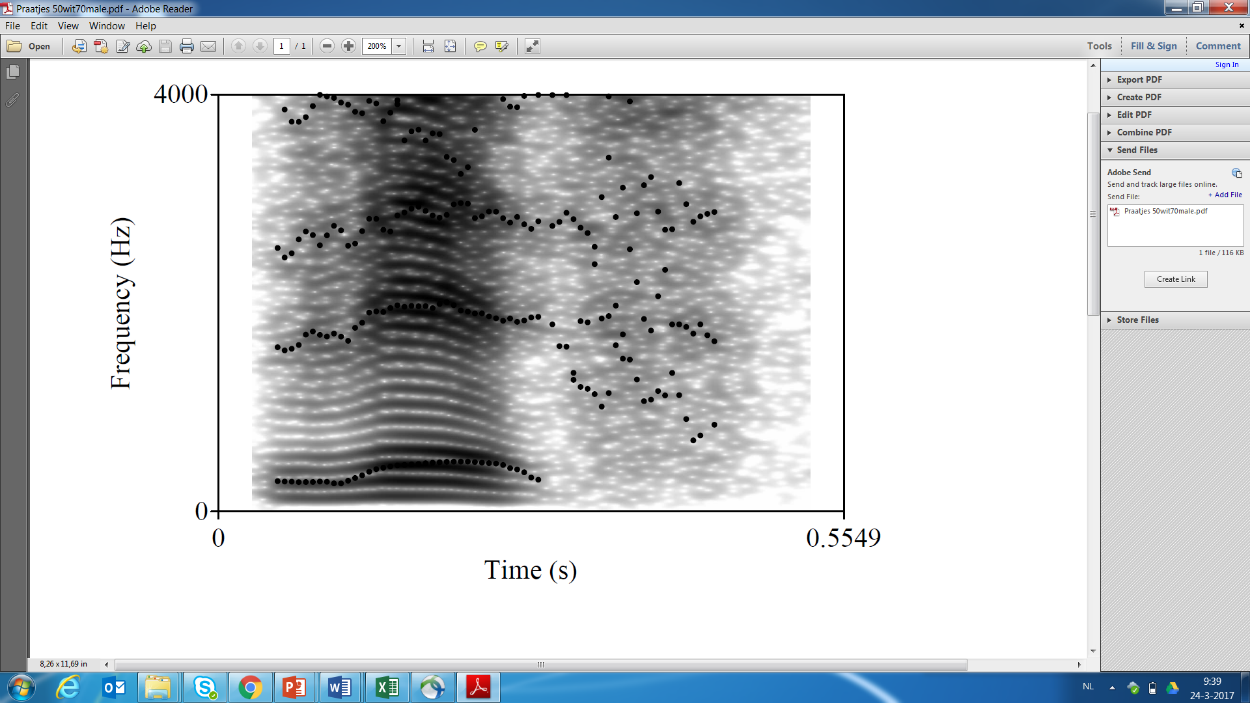
G. Tr7 H. Tr8


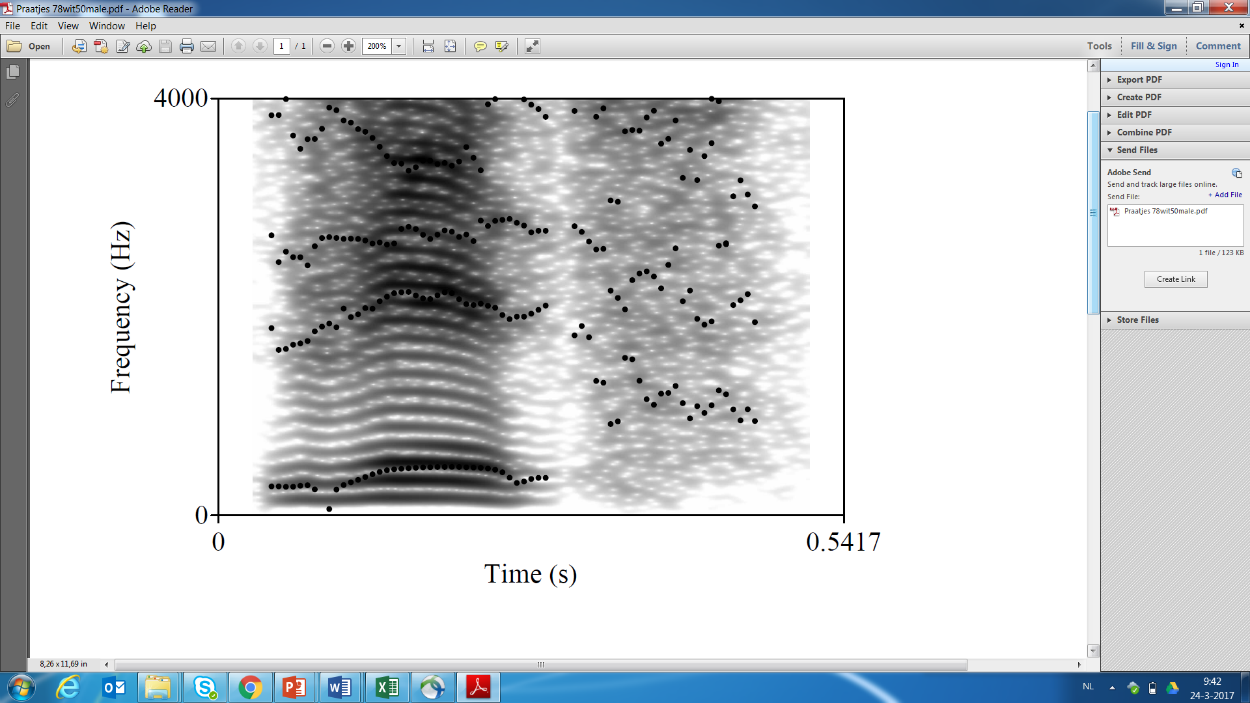
**Fig 3** Spectrograms and the formant trajectories for training sounds (E. Tr5; F. Tr6; G. Tr7; H. Tr8) for the diagonal mapping from set 1

**Fig 4** Vowel chart of the first four training sounds from three sets. Frequencies in Hz of the first and second formants of Tr1, Tr2, Tr3 and Tr4 of each set are plotted against each other

**Table 2** Mean proportion of correct responses (plus standard deviation) for birds in the four categorization mappings. The within-category intermediate sound type is indicated with ‘intermediate’

| Soundgroup | Vowel | Speaker sex | Diagonal | XOR |
| --- | --- | --- | --- | --- |
| Trained | 0.87 ± 0.09 | 0.89 ± 0.04 | 0.87 ± 0.05 | 0.86 ± 0.06 |
| Extreme | 0.61 ± 0.06 | 0.78 ± 0.08 | 0.74 ± 0.06 | 0.54 ± 0.05 |
| Ambiguous | 0.58 ± 0.04 | 0.62 ± 0.05 |  | 0.55 ± 0.03 |
| Intermediate | 0.62 ± 0.10 | 0.80 ± 0.05 | 0.68 ± 0.08 |  |

**Table 3** Mean proportion of correct responses (plus standard deviation) for humans in the four categorization mappings. The within-category intermediate sound type is indicated with ‘intermediate’

| Soundgroup | Vowel | Speaker sex | Diagonal | XOR |
| --- | --- | --- | --- | --- |
| Trained | 0.91 ± 0.09 | 0.83 ± 0.16 | 0.73 ± 0.09 | 0.68 ± 0.19 |
| Extreme | 0.89 ± 0.14 | 0.86 ± 0.16 | 0.92 ± 0.09 | 0.71 ± 0.20 |
| Ambiguous | 0.82 ± 0.12 | 0.72 ± 0.11 |  | 0.60 ± 0.15 |
| Intermediate | 0.91 ± 0.12 | 0.83 ± 0.17 | 0.82 ± 0.10 |  |

**Table 4** Mean proportion of correct responses on vowel test for individual birds. The within-category intermediate sound type is indicated with ‘intermediate’

| Bird ID | Trained | Extreme | Ambiguous | Intermediate |
| --- | --- | --- | --- | --- |
| 341 | 0.85 | 0.64 | 0.59 | 0.57 |
| 360 | 0.76 | 0.57 | 0.59 | 0.65 |
| 368 | 0.85 | 0.57 | 0.57 | 0.61 |
| 539 | 0.94 | 0.62 | 0.61 | 0.49 |
| 547 | 0.93 | 0.63 | 0.64 | 0.72 |
| 550 | 0.68 | 0.52 | 0.49 | 0.48 |
| 557 | 0.99 | 0.76 | 0.62 | 0.76 |
| 585 | 0.92 | 0.62 | 0.57 | 0.73 |
| 644 | 0.94 | 0.57 | 0.57 | 0.62 |


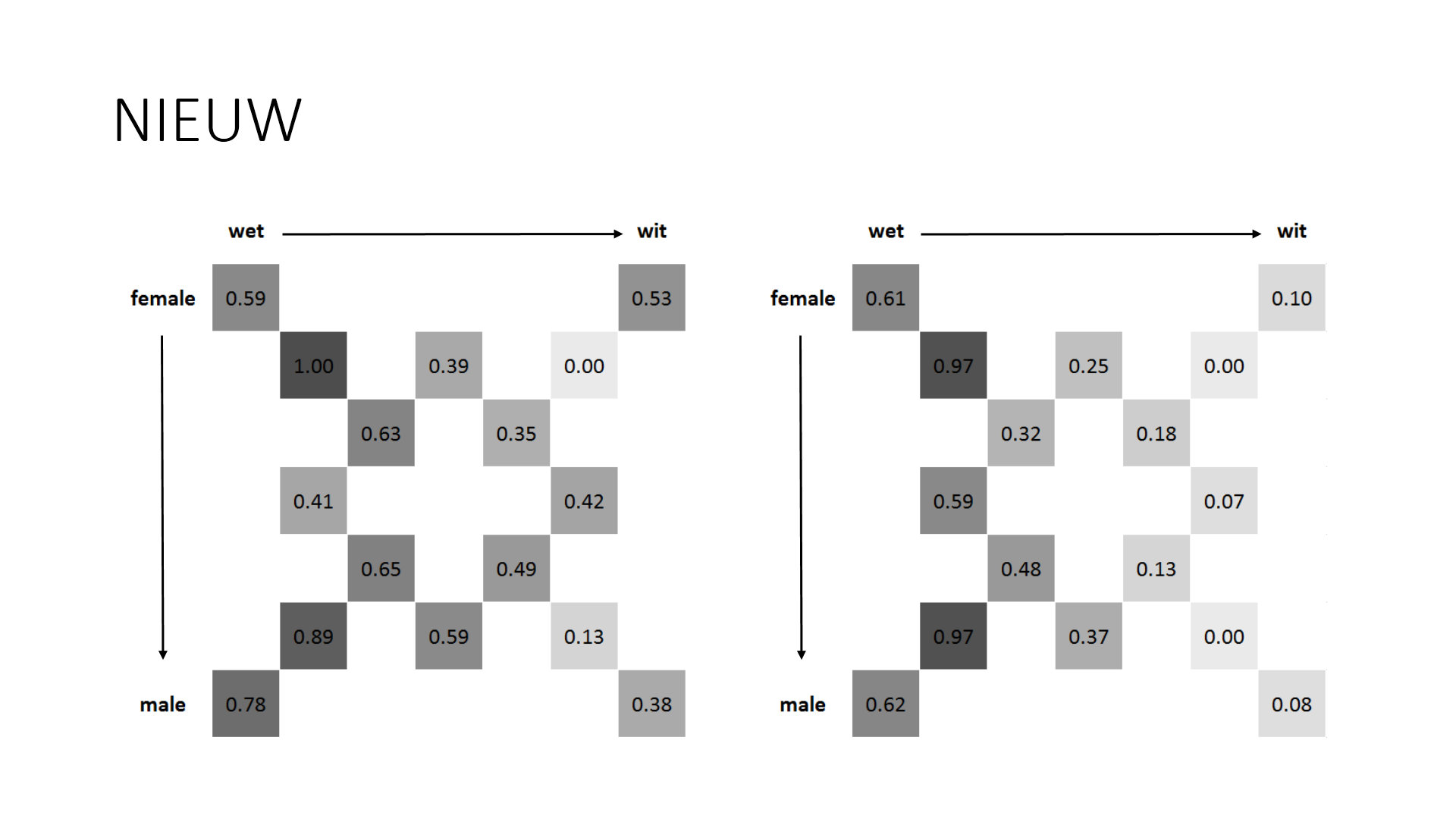


**Fig 5** Mean proportion of *wet* responses (high= dark grey, low = light grey) for two individual birds in the vowel test. A high score reflects that the bird classified the sound as *wet*; a low score reflects that the bird classified the sound as *wit*. Left: for this bird (bird ID 539), the responses are consistently high for the two trained *wet* sounds and consistently low for two trained *wit* sounds. This bird shows a less consistent response pattern for the new test-sounds. This profile suggests exemplar memorization. Right: A different bird (bird ID 557) showing consistently high responses for the two trained *wet* sounds and consistently low for two trained *wit* sounds. This bird shows substantially higher scores for all test-sounds on the *wet* side than on the *wit* side, suggesting generalization to new test-sounds indicative of (partial) rule learning

**Table 5** Mean proportion of correct responses on speaker sex test for individual birds. The within-category intermediate sound type is indicated with ‘intermediate’

| Bird ID | Trained | Extreme | Ambiguous | Intermediate |
| --- | --- | --- | --- | --- |
| 315 | 0.95 | 0.64 | 0.62 | 0.80 |
| 318 | 0.87 | 0.89 | 0.66 | 0.84 |
| 431 | 0.93 | 0.87 | 0.68 | 0.89 |
| 437 | 0.92 | 0.82 | 0.57 | 0.83 |
| 471 | 0.80 | 0.70 | 0.54 | 0.72 |
| 496 | 0.87 | 0.72 | 0.64 | 0.76 |
| 511 | 0.89 | 0.78 | 0.63 | 0.73 |
| 551 | 0.94 | 0.79 | 0.72 | 0.83 |
| 361 | 0.94 | 0.89 | 0.60 | 0.84 |


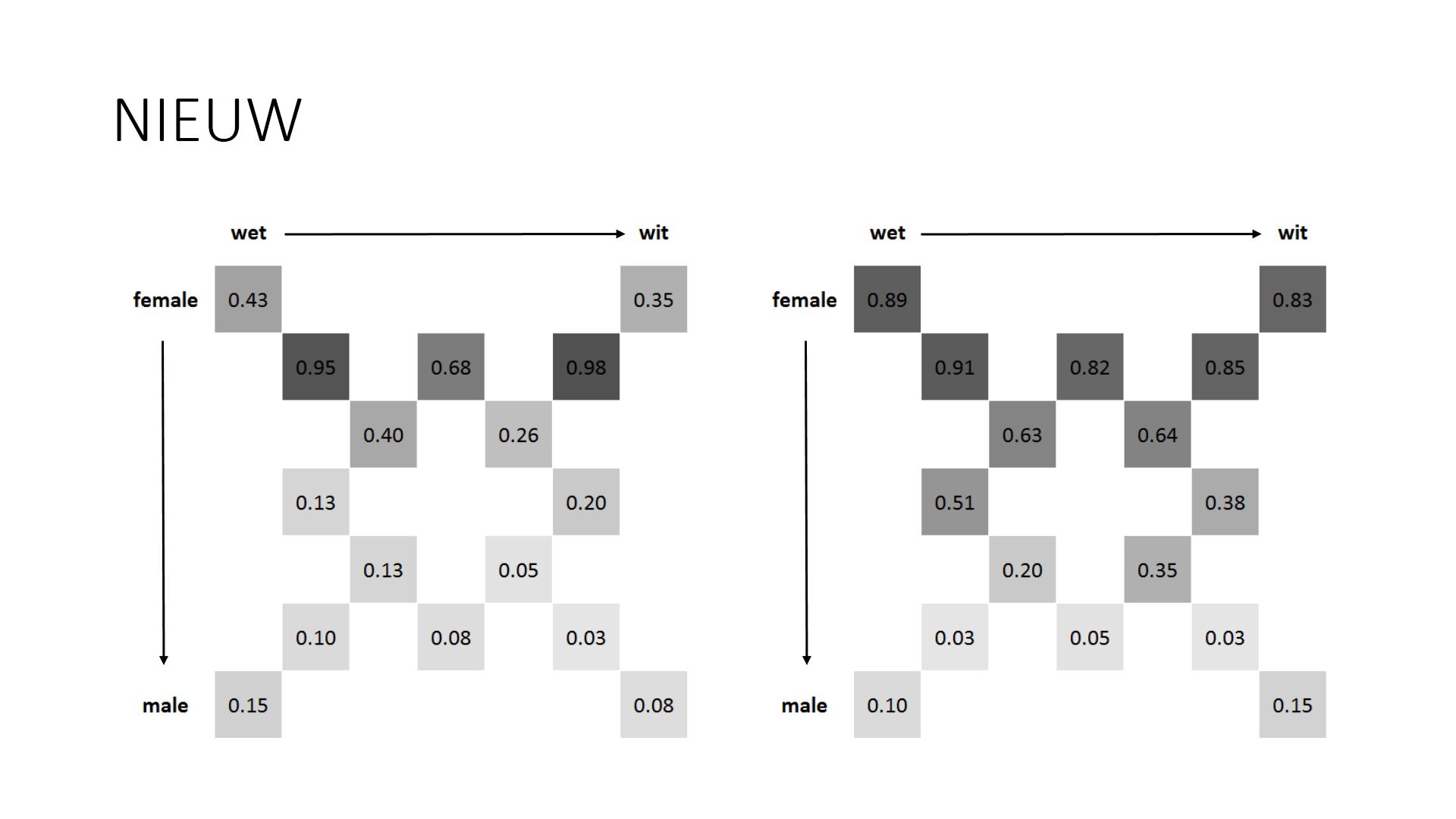


**Fig 6** Mean proportion of female responses (high= dark grey, low = light grey) for two individual birds in the speaker sex test. A high score reflects that the bird classified the sound as female; a low score reflects that the bird classified the sound as male. Left for this bird (bird ID 315), the responses are consistently high for the two trained female sounds and consistently low for two trained male sounds. This birds shows a less consistent response pattern for the new test-sounds, although some generalization, in particular to the ‘male’ side, is present. Right A different bird (bird ID 431) showing consistently high responses for the two trained female sounds and consistently low for two trained male sounds. Furthermore, the responses of this bird to the intermediate and extreme test-sounds are comparable to the responses to the training sounds. This bird thus shows generalization to new test-sounds, indicative of rule learning

**Table 6** Mean proportion of correct responses on diagonal test for individual birds. The within-category intermediate sound type is indicated with ‘intermediate’

| Bird ID | Trained | Extreme | Intermediate |
| --- | --- | --- | --- |
| 470 | 0.82 | 0.84 | 0.72 |
| 472 | 0.90 | 0.65 | 0.79 |
| 473 | 0.92 | 0.79 | 0.72 |
| 474 | 0.92 | 0.86 | 0.72 |
| 538 | 0.87 | 0.74 | 0.56 |
| 666 | 0.86 | 0.71 | 0.62 |
| 489 | 0.80 | 0.70 | 0.55 |
| 493 | 0.81 | 0.70 | 0.78 |
| 662 | 0.94 | 0.76 | 0.74 |


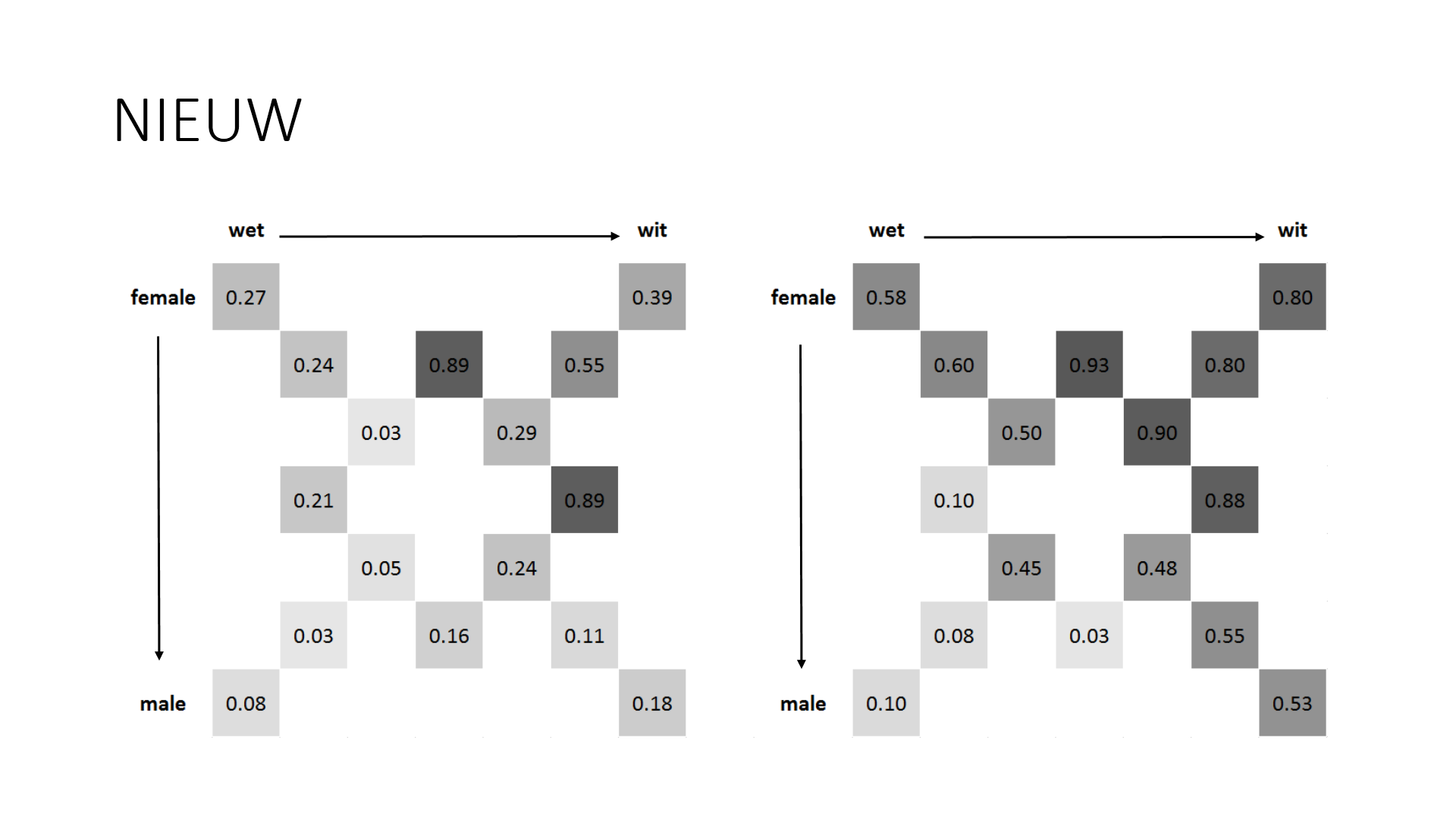


**Fig 7** Mean proportion of responses for the female *wit* like sounds (high= dark grey, low = light grey) for two individual birds in the diagonal test. A high score reflects that the bird classified the sound as more female *wit* like; a low score reflects that the bird classified the sound as more male *wet* like. Left for this bird (bird ID 666), the responses to one sensor are consistently high for the two trained sounds on the female ‘wit’ side and consistently low for two trained sounds on the male *wet* side. This birds shows a less consistent response pattern for the new test-sounds. This bird thus shows strong exemplar memorization. Right for this bird (bird ID 474), the responses to one sensor are consistently high for the two trained sounds on the female *wit* side and consistently low for two trained sounds on the male *wet* side. This bird gives similar responses to extreme sounds and to one of the intermediate sounds. This bird thus shows generalization to new test-sounds

**Table 7** Mean proportion of correct responses on XOR test for individual birds

| Bird ID | Trained | Extreme | Ambiguous |
| --- | --- | --- | --- |
| 350 | 0.90 | 0.52 | 0.58 |
| 358 | 0.70 | 0.63 | 0.51 |
| 384 | 0.83 | 0.56 | 0.52 |
| 497 | 0.82 | 0.46 | 0.54 |
| 505 | 0.94 | 0.60 | 0.60 |
| 510 | 0.90 | 0.57 | 0.55 |
| 512 | 0.90 | 0.53 | 0.56 |
| 588 | 0.92 | 0.53 | 0.54 |
| 643 | 0.88 | 0.49 | 0.61 |
